# Leveraging gene correlations in single cell transcriptomic data

**DOI:** 10.1101/2023.03.14.532643

**Published:** 2023-03-15

**Authors:** Kai Silkwood, Emmanuel Dollinger, Josh Gervin, Scott Atwood, Qing Nie, Arthur D. Lander

**Affiliations:** 1 Center for Complex Biological Systems, University of California, Irvine, Irvine CA.; 2 Department of Developmental and Cell Biology, University of California, Irvine, Irvine CA.; 3 Department of Mathematics, University of California, Irvine, Irvine CA.

**Keywords:** single cell RNA sequencing, gene-gene correlation, gene regulatory network, gene co-expression network, melanoma

## Abstract

Many approaches have been developed to overcome technical noise in single cell (and single nucleus) RNA-sequencing (scRNAseq). As researchers dig deeper into data—looking for rare cell types, subtleties of cell states, and details of gene regulatory networks—there is a growing need for algorithms with controllable accuracy and a minimum of *ad hoc* parameters and thresholds. Impeding this goal is the fact that an appropriate null distribution for scRNAseq cannot simply be extracted from data in the event that ground truth about biological variation is unknown (i.e., most of the time). Here we approach this problem analytically, based on the assumption that scRNAseq data reflect only cell heterogeneity (what we seek to characterize), transcriptional noise (temporal fluctuations randomly distributed across cells), and sampling error (i.e., Poisson noise). We then analyze scRNAseq data without normalization—a step that can skew distributions, particular for sparse data—and calculate *p*-values associated with key statistics. We develop an improved method for the selection of features for cell clustering and the identification of gene-gene correlations, both positive and negative. Using simulated data, we show that this method, which we call BigSur (Basic Informatics and Gene Statistics from Unnormalized Reads), accurately captures even weak yet significant correlation structures in scRNAseq data. Applying BigSur to data from a clonal human melanoma cell line, we identify tens of thousands of correlations that, when clustered without supervision into gene communities, both align with cellular components and biological processes, and point toward potentially novel cell biological relationships.

## Introduction

Single cell RNA-sequencing (scRNAseq), along with the related method of single nucleus RNA-sequencing, now offers researchers unparalleled opportunities to interrogate cells as individuals. Methods have been developed to classify cell types; identify gene expression markers; infer lineages; learn gene regulatory relationships, and examine the effects of experimental manipulations on both levels of gene expression and cell type abundances (Das et al., 2022; Junttila et al., 2022; Nguyen et al., 2021; Simmons, 2022; Tam and Ho, 2020; Tritschler et al., 2019; Xie et al., 2021). Because scRNAseq data are noisy, reliable inference requires leveraging information across many cells, trading off sensitivity for statistical power. How to handle that tradeoff should depend, ideally, on one’s goal. Simply clustering large numbers of transcriptionally very different cells (“cell types”) into small numbers of groups of similar size allows for a great deal of latitude in aggregating information across cells; it is thus not surprising that many different clustering approaches all perform well. Other tasks, such as ordering cells along a continuum of gene expression change, or picking out rare cell populations within much larger groups, are less forgiving, and a plethora of different approaches currently compete for investigators’ attention (Bej et al., 2021; Bocci et al., 2022; Dann et al., 2022; Dong and Yuan, 2020; Gerniers et al., 2021; Grun et al., 2015; Herman et al., 2018; Jiang et al., 2016; Jin et al., 2021; Jindal et al., 2018; Lange et al., 2022; Setty et al., 2019; Sha et al., 2020; Wang and Ma, 2022; Wegmann et al., 2019; Zhang and Nie, 2021). Assessing the performance of such methods is frequently hindered by the absence of ground truth.

A particularly challenging application of scRNAseq is in the identification of patterns of gene co-expression. The identification of large-scale blocks of co-expressed genes—co-expression “modules”—can provide an alternative method for classifying cells when traditional clustering fails (Bageritz et al., 2019). In contrast, smaller-sized blocks of gene co-expression have the potential to reflect true gene-regulatory networks that relate to specific functions (Kolodziejczyk et al., 2015). This is because random transcriptional noise in gene circuits should induce weak but real correlations among regulatory genes and their targets. Indeed, it has long been proposed that gene regulatory links could be discovered solely from the weak gene expression correlations that one might encounter when studying otherwise homogenous populations of cells (Becskei et al., 2005; Gupta et al., 2022; Munsky et al., 2012; Padovan-Merhar and Raj, 2013; Pedraza and van Oudenaarden, 2005; Stewart-Ornstein et al., 2012; Warmflash and Dinner, 2008).

Unfortunately, identifying small yet significant gene expression correlations in single cell data requires a degree of statistical power that scRNAseq applications rarely strive for (and, to be fair, rarely need to). Yet, as greater numbers of scRNAseq datasets accumulate, with a growing trend toward increasing numbers of cells per dataset, we wondered whether substantial amounts of novel information about gene co-regulation might be accessible simply through a more in-depth examination of pairwise gene expression correlations.

One of the main challenges in pursuing such a program is the absence of an accepted statistical model for pairwise correlations in scRNAseq data. Only with a model can one define a null hypothesis by which to judge whether observations are significant. Unfortunately, with scRNAseq, there is not good consensus regarding the model to use for the data distributions of individual genes, much less their correlations. The common approach of fitting individual gene data to *ad hoc* analytical distributions (e.g. “zero-inflated negative binomial” (He et al., 2021); reviewed by (Choudhary and Satija, 2022)), has met with frequent criticism that is difficult to dismiss (Choi et al., 2020; Kim et al., 2015; Sarkar and Stephens, 2021; Wang et al., 2018). One may seek to circumvent such concerns by attempting to learn empirical distributions on a case-by-case basis from data, but this typically requires making assumptions about the amount of actual biological variation in the data, which is frequently unknown. Furthermore, pitfalls in implementing empirical methods can be hard to avoid, particularly with high-order statistical information, such as correlations. For example, the seemingly reasonable intuition that one might be able to construct the distribution of the correlation coefficient under the null hypothesis simply by randomly permuting elements is actually incorrect (DiCiccio and Romano, 2017).

One of the major obstacles in defining an appropriate data distribution for scRNAseq data is the fact that underlying sources of technical variation are not fullly understood, nor is the range of biological variation in biologically “equivalent” cells fully known. Here we begin by re-considering these factors, and leveraging the work of others, in pursuit of an analytical model of null correlation distributions that makes the fewest ad hoc assumptions and minimizes adjustable parameters. We show that the approach that emerges has the power to identify subtle yet real correlations, both positive and negative, in scRNAseq data, even among genes that are relatively sparsely sequenced. Ultimately this method should be applicable not only to the identification of gene regulatory interactions, but to more complex tasks based on gene-gene correlations—such as the identification of cellular trajectories (Jin et al., 2018) and “tipping points” (Yang et al., 2022)—as well as providing a means to achieve a more principled approach to basic, early steps in scRNAseq analysis—such as normalization, batch correction, feature selection and clustering.

## Results

### The significance of gene-gene correlations

The statistical significance of correlations is rarely discussed because, for many common kinds of data—those that are continuous and at least approximately normal in distribution—the magnitude of correlation and its significance are related in a simple way that depends only on the number of measurements, and not the data distributions. Owing to Fisher (Fisher, 1950), for any Pearson correlation coefficient (PCC), the *p*-value (probability of observing |PCC|≥*x* by chance) may be estimated as $\text{Erfc}\left[\sqrt{\left(n-3\right)/2\;}\text{arctanh}\left(\left|x\right|\right)\right]$, where *n* is the number of samples, and *Erfc* is the complement of the Error function (we refer to this expression henceforth as the “Fisher formula”).

**Figure 1.**
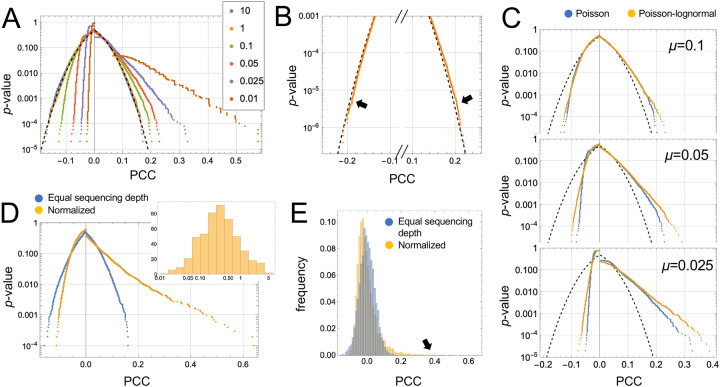
Relationship between Pearson correlation coefficient, vector sparsity and *p*-value. The panels compare *p*-values determined empirically by correlating 50,000 pairs of random, independent vectors of length 500 with *p*-values predicted by the Fisher formula. **A.** Data were independent random variates from Poisson distributions with means as indicated. The dashed line shows the output of the Fisher formula. **B.** Even for vectors drawn from a distribution with mean = 1, the Fisher formula significantly mis-estimated p-values smaller than 10^−4^. **C.** Poor performance of the Fisher formula is worsened when data are drawn from a Poisson-log-normal distribution, rather than a Poisson distribution (in this case the underlying log-normal distribution had a coefficient of variation of 0.5). **D-E.** Data were simulated under the scenario that gene expression is the same in every cell, but due to differences in sequencing depth, observed gene expression varies according to the depths shown in the inset to panel D. In panel D, true gene expression was adjusted so that observed gene expression after normalization would have a mean of 1, and the Pearson correlation coefficients (PCCs) obtained by correlating randomly chosen vectors are shown. Panel D plots empirically-derived *p*-values as a function of PCC, whereas panel E displays histograms of PCCs. Compared with data that do not require normalization, associated *p*-values from normalized data are even more removed from the predictions of the Fisher formula.

Because scRNAseq data are both discrete and generally not normally distributed, use of the Fisher formula is not justified, but just how far off will its results be? Figure 1 explores that question through simulation. Assuming Poisson-distributed data, and gene expression vectors of 500 cells in length, the formula does poorly for vectors of mean < 1, i.e., where the expected proportion of zeros exceeds 37%, assigning *p*-values that are too low for positive correlations, and too high for negative ones (Fig. 1A). For distributions with mean ≥1 (fewer than 37% zeros on average), the formula does reasonably well down to *p*-values as low as 10^−4^ but deviates progressively thereafter. This would clearly be a problem for any genome-wide analysis of correlations: To analyze pair-wise correlations among *m* genes one must test *m*(*m*-1)/2 hypotheses. With values of *m* often ≥ 12,000, this amounts to >7 × 10^8^ simultaneous tests, such that statistical significance of any single observation could potentially require *p* as low as 10^−9^, a value for which we may estimate, by extrapolation, that the Fisher formula is highly inaccurate even for genes with mean expression = 1.

The simulations in Fig 1A–B assume Poisson-distributed data but, as is often pointed out, scRNAseq data are usually over-dispersed relative to the Poisson distribution (more on this below). As shown in Fig. 1C, adding in such additional variance causes simulated data to deviate even further from the Fisher formula.

Further problems arise when considering that scRNAseq data always come from collections of cells with widely varying total numbers of UMI. Depending on the platform, such “sequencing depth” can vary over orders of magnitude, which is why normalization is usually a critical early step in data analysis. Without normalization, it is obvious that many spurious gene-gene correlations would be detected, as any difference in sequencing depth between cells would, if not corrected for, induce positive correlation across all genes.

As one might expect, normalizing individual reads by scaling them to each cell’s sequencing depth eliminates this bias, restoring the expected value of PCC under the null hypothesis to zero. Yet normalization does not restore the *distribution* of PCCs to what it would have been had all cells been sequenced equally. The consequences can be dramatic, as shown in Fig. 1D–E, where we simulate a case in which “true” gene expression is the same in each of 500 cells, but observed gene expression is a Poisson random variate from a mean that was scaled by a factor chosen from a distribution of cell-specific sequencing depths similar to what one might observe in a typical scRNAseq experiment, using the 10X Chromium platform (inset, Fig. 1D). Despite mean gene expression being ~1, the relationship between PCC and *p*-value more closely resembles the case (in the absence of sequencing depth variation) where the mean is 0.01 (Fig. 1A). This is surprising, given that the average fraction of zeros in the normalized vectors was only 0.68 (which, for Poisson-distributed data, would be expected at a mean expression level of 0.39). In short, for gene expression data resembling what is typically obtained in scRNAseq, the Fisher formula is highly unsuitable, for most pairs of genes, for estimating the significance of correlations.

### An analytical model for the distribution of correlations

The data in Figure 1 indicate that the relationship between PCCs and *p*-values is highly sensitive both to data structure and procedures intended to “correct” for technical variation. Because of this, we were concerned that a suitable null model for the distribution of correlations might be difficult to estimate empirically, especially when the true biological variation in most datasets is unknown. We therefore turned to constructing a null model analytically, attempting to account for known sources of variation (beside meaningful biological variation). The three sources we considered were (1) variation introduced by imperfect normalization; (2) technical variation due to random sampling of transcripts during library preparation and sequencing; and (3) variation due to stochasticity of gene expression. The last of these is not truly “technical” variation—fluctuating gene expression is a biological phenomenon—but like technical noise, gene expression fluctuation is usually an unwanted source of variation, and its effects need somehow to be suppressed.

With regard to the first source, we follow (Lause et al., 2021) in correcting not the gene expression data points themselves, but rather their Pearson residuals. Traditionally, the Pearson residual *P*_*ij*_ for cell *i* and gene *j*, is defined as

$$P_{ij}=\frac{x_{ij}-\mu_{j}}{\sqrt{\mu_{j}}}$$

where *x*_*ij*_ is the gene expression value for cell *i* and gene *j*, and *μ*_*j*_ is the mean expression of gene *j* averaged over all cells. As the Pearson residual is mean-centered, its expectation value, *E*[*P*_*ij*_], is zero; thus, the average of Pearson residuals for a large number of *x*_*ij*_ drawn from a single distribution should approach zero. The average of squares of Pearson residuals can be seen, by inspection, to approach the variance divided by the mean of the distribution from which the *x*_*ij*_ derive. Variance divided by mean is also known as the Fano factor and is often used to assess whether data are consistent with a Poisson distribution since, for any Poisson distribution regardless of mean, *E*[*P*_*ij*_^2^] = 1.

Pearson residuals may also be used to construct PCCs, which are commonly defined in terms of variances and covariances, but may be equivalently expressed as:

$${\text{PCC}}_{a\times b}=\frac{\sum_{i=1}^{n}P_{ia}P_{ib}}{\sqrt{\sum_{i=1}^{n}{P_{ia}}^{2}\sum_{i=1}^{n}{P_{ib}}^{2}}}=\frac{1}{\left(n-1\right)\sqrt{\phi_{a}\phi_{b}}}\sum_{i=1}^{n}P_{ia}P_{ib}$$

where PCC_*a*×*b*_ is the Pearson correlation coefficient between genes *a* and *b*, *n* the number of cells, and *ϕ*_*a*_ and *ϕ*_*b*_ represent the Fano factors for genes *a* and *b*, respectively, i.e.


$$\phi_{j}=\frac{1}{n-1}\sum_{i=1}^{n}{P_{ij}}^{2}$$

Since *E*[*P*_*ij*_] = 0 for any gene, it follows that *E*[PCC_*a*×*b*_] = 0, as long as all the expression values for gene *a* are drawn from a single distribution, and those for *b* are independently drawn from a single distribution.

However, in scRNAseq, unequal sequencing depth means that the expression values for any given gene are generally not drawn from a single distribution, but rather from one that is different for each cell. Interestingly, dividing *x*_*ij*_ in a Pearson residual by an appropriate scaling constant—i.e. normalizing the data—will restore *E*[*P*_*ij*_] to 0, but will not restore the higher moments of *P*_*ij*_, e.g., *E*[*P*_*ij*_^2^] ≠ 1. To capture the correct second moment, one must scale the value of *μ*_*j*_ inside each Pearson residual, rather than scaling *x*_*ij*_. As (Lause et al., 2021) have pointed out, we can define a separate *μ*_*ij*_ for each cell and gene:

$$\mu_{\text{ij}}=\frac{\sum_{j}x_{\text{ij}}\sum_{i}x_{\text{ij}}}{\sum_{ij}x_{\text{ij}}}$$

and consequently, define a corrected Pearson residual as

$$P_{i,j}=\frac{x_{\text{ij}}-\mu_{\text{ij}}}{\sqrt{\mu_{\text{ij}}}}$$

Although this transformation does not recover moments of the Pearson residual beyond the first two, it provides a principled alternative to traditional normalization. Moreover, by permitting calculation of a corrected Fano factor that has the appropriate expectation value under the assumption of Poisson distributed data, it can be used to test that assumption, in real data.

Source #2 refers to technical variation due to random, independent sampling of discrete numbers of transcripts. Although the sampling process in scRNAseq involves several discrete steps, including cell lysis, library preparation and DNA sequencing, several groups have argued that, at least at modest to low expression levels, simple Poisson “noise” can reasonably model the variation derived from these processes (Choi et al., 2020; Kim et al., 2015; Sarkar and Stephens, 2021; Wang et al., 2018). We accept this assumption here, but note that, in the following derivations, the Poisson distribution could just as easily be replaced by another known distribution, if it were adequately justified.

Finally, source #3 refers to the fact that “equivalent” cells are usually only equivalent in a time-averaged sense, i.e., transcript numbers will fluctuate around some mean value. Both theory and observation support the conclusion that these fluctuations can be large (Bahar Halpern et al., 2015; Munsky et al., 2012; Schwabe et al., 2012; Thattai and van Oudenaarden, 2001). The actual magnitude seems to differ for different categories of genes, but data from single-molecule transcript counting (Bahar Halpern et al., 2015) suggest that, for most genes, the distribution of transcripts typically is approximately log-normal (consistent with the theoretical work of (Beal, 2017)), with a coefficient of variation in the range of 0.2–0.6.

Thus, even if library synthesis and sequencing perform identically across cells, one should not expect to obtain a distribution of reads fitting a Poisson distribution. Under the reasonable assumption that gene expression fluctuations and sampling are independent, the variance of the combined process should be the sum of the variances of the composing processes. Since both processes have the same mean, we can re-state this as the Fano factor of the combined process should be the sum of the Fano factors of the composing processes. One can then use this fact to further “correct” the Pearson residual. Specifically, if we define a “modified corrected Pearson residual” as:

(Eq. 1) $$P_{\text{ij}}\prime =\frac{x_{ij}-\mu_{ij}}{\sqrt{\mu_{ij}\left(1+{c_{j}}^{2}\mu_{ij}\right)}}$$

where *c* represents the coefficient of variation of gene expression for gene *j*, then the expectation value for (*P*_ij_′)^2^

$$\frac{<\frac{{\left(x_{ij}-\mu_{ij}\right)}^{2}}{\mu_{ij}}>}{\left(1+{c_{j}}^{2}\mu_{\text{ij}}\right)}$$

resembles a Fano factor divided by (1 + *c*_*j*_^2^*μ*_ij_). Since *c*^2^*μ*_ij_ can alternatively be written as $\frac{\sigma_{\text{ij}}}{\mu_{\text{ij}}}$ where *σ* is the standard deviation of gene expression fluctuations, the term (1 + *c*_*j*_^2^*μ*_ij_) is simply the sum of the Fano factors for Poisson sampling (unity) and gene expression fluctuation (*c*_*j*_^2^*μ*_ij_). Dividing by that sum essentially removes the additional variance due to gene expression noise from the expectation value of (*P*_ij_′)^2^, restoring that value to 1.

In this way, one may define a “modified corrected Fano factor” *ϕ*′ equal to the expectation value of (*P*_ij_′)^2^, and a modified corrected Pearson correlation coefficient, PCC′:

(Eq. 2) $$\text{PC}{{\text{C}}^{\prime }}_{a\times b}=\frac{1}{\left(n-1\right)\sqrt{\phi \prime_{a}\phi \prime_{b}}}\sum_{i=1}^{n}P\prime_{ia}P\prime_{ib}$$

In analyzing scRNAseq, *ϕ*′ provides a measure of the degree to which a gene’s expression is more variable than expected by chance, and PCC′ provides a metric by which gene-pairs are more positively or negatively correlated than expected by chance, correcting in both cases for unequal sequencing across cells (without normalizing data) and the expected noisiness of gene expression.

Of course, to use these statistics in practice, one needs to know not only their expectation values but also their full distributions under the null hypothesis. Constructing those analytically requires not only the coefficient of variation of gene expression noise, *c*, but the full distribution of that noise, which in the absence of information to the contrary, we will take to be log-normal (Beal, 2017), noting that any other distribution could as easily be substituted in the following discussion.

We thus treat *x*_*ij*_ as a Poisson random variable drawn from a distribution whose mean is a log-normal random variable and whose coefficient of variation is *c* (we refer to this compound distribution as Poisson-log-normal). Although an analytical form for the probability mass function of the Poisson-log-normal distribution is not known, we may derive analytical forms for an arbitrary number of its moments, as a function of *μ* (the mean) and *c* (see Methods). Thus, one can calculate for every cell *i* and gene *j*, given the observed value of *μ*_ij_, the moments of the expected distributions of the *P*_ij_′ under the null hypothesis, and subsequently those for the (*P*_ij_′)^2^. From there one can calculate the moments of any number of products and sums of *P*_ij_′ and (*P*_ij_′)^2^, such that, eventually, the moments of *ϕ*′ and PCC′ under the null hypothesis are ultimately obtained. Given a finite number of moments, one can estimate the tails of the distributions of these statistics (see Methods), allowing one to calculate the probability of extreme values of *ϕ*′ and PCC′ arising by chance (*p*-values).

This method, which we refer to as BigSur (Basic Informatics and Gene Statistics from Unnormalized Reads), provides an approach for discovering genes that are significantly variable across cells (based on *ϕ*′), and gene pairs that are significantly positively or negatively correlated (based on PCC′), automatically accounting for the widely varying distributions of these statistics as a function of mean gene expression level and vector length (number of cells). The one free parameter in the method, *c*, is relatively constrained, as its average value (over all genes) can be estimated from a plot of *ϕ*′ versus mean expression (see below). In this manner, one can avoid the use of any arbitrary thresholds or cutoffs in deciding which genes are significantly “highly” variable (e.g., for dimensionality reduction and cell classification) and which genes are significantly positively and negatively correlated (e.g., to discover gene expression modules and construct regulatory networks).

### Performance on simulated data

**Figure 2.**
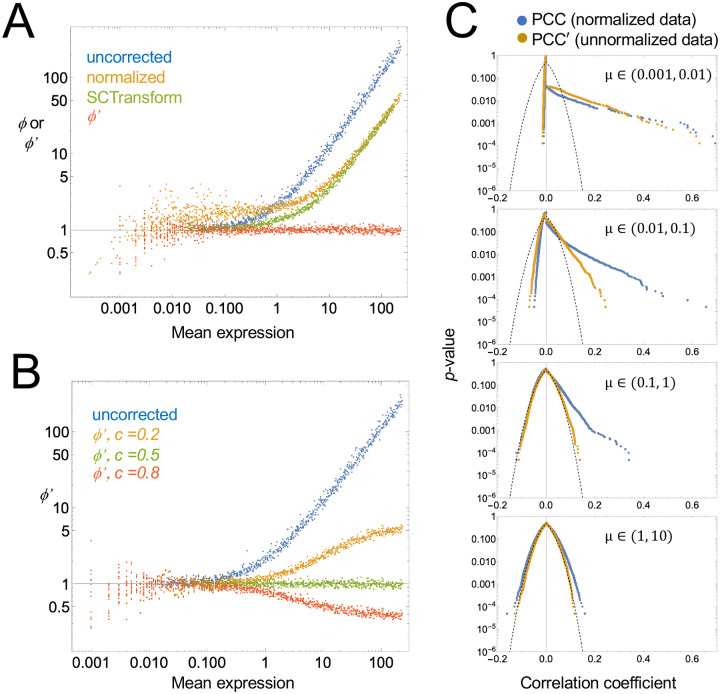
**Comparing uncorrected and modified corrected Fano factors and correlation coefficients.** Random, independent, uncorrelated gene expression data was generated for 1000 genes in 999 cells, under the assumption that observations are random Poisson variates from a per-cell expression level that is itself a random variate of a log-normal distribution, scaled by a sequencing depth factor that is different for each cell (see methods). **A.** Uncorrected (*ϕ*) or modified corrected (*ϕ*′) Fano factors are plotted as a function of mean expression level for each gene. Uncorrected factors were calculated either without normalization, or with default normalization (scaling observations by sequencing depth factors, learned by summing the gene expression in each cell). Uncorrected Fano factors were also calculated using SCTransform (Hafemeister and Satija, 2019) as an alternative to default normalization. Modified corrected Fano factors were obtained by applying BigSur to unnormalized data, using a coefficient of variation parameter of *c*=0.5. **B.** Modified corrected Fano factors (*ϕ*′) were calculated as in A, but using different values of *c*. The data suggest that an optimal choice of *c* can usually be found by examining a plot of *ϕ*′ versus mean expression. **C.** Empirical *p*-values associated with uncorrected (PCC) or modified corrected (PCC′) Pearson correlation coefficients were calculated for pairwise combinations of genes in bins of different mean gene expression level (*μ*); examples are shown for four representative bins (both genes derived from the same bin). With increasing gene expression levels the *p*-value vs. PCC relationship begins to approach the Fisher formula (dashed curve), but it does so much sooner for PCC′ than PCC.

In Figure 2 we simulate gene expression data for 1000 genes and 999 cells, under the null model described above (i.e., complete independence), varying “true” mean expression uniformly over the genes, such that the most highly expressed average 3467 transcripts/cell and the most lowly expressed 0.0351 per cell. “Observed” gene expression values are then obtained by randomly sampling from a Poisson log-normal distribution with *c*=0.5, in which the gene-specific mean is first scaled in each cell according to a pre-defined distribution of sequencing depth factors (chosen to mimic typical ranges of sequencing depth when using the 10X Chromium platform). The result is a set of gene expression vectors of length 999, with means varying between 0.001 and 231.

As shown in Figure 2, for most genes with mean expression greater than ~0.1 URM/cell, uncorrected Fano factors exceed 1, and rise linearly with expression level. Normalizing the data—scaling expression in each cell to the relative sequencing depth of that cell—reduces high-expression skewing somewhat, but also elevates the Fano factors for genes with low expression, driving them closer to 2. These results, in which the majority of genes display Fano factors greater than 1, which rise further for highly expressed genes, agree with the pattern most commonly seen in actual scRNAseq data. Values of the Fano factor for lowly expressed genes may be restored to near 1 by normalizing using SCTransform, an algorithm designed to correct for some of the variance-inflating aspects of normalization-by-scaling (Hafemeister and Satija, 2019), but the presence of high Fano factors among the highly expressed genes persists.

In contrast, if we calculate the modified corrected Fano factor, *ϕ*′, for each gene, using *c*=0.5, we see that values are now centered around 1 at all expression levels (Fig. 2A). However, if we choose other values of *c*, we observe either positive or negative skewing at mean gene expression values above 1 (Fig. 2B). Under the assumption that most genes in real data should not vary significantly across cells, one may therefore estimate *c* for any data set by simply finding the value that minimizes such high-expression skewing of *ϕ*′.

Because it uses the moments of the Pearson residuals to calculate *p*-values, BigSur assigns statistical significance to every gene’s *ϕ*′. As expected, given that the data were random samples, no values of *ϕ*′ in Fig. 2A were found to be statistically significant at a *p*-value threshold of 0.05 (corrected for multiple sampling), or a Benjamini-Hochberg (Benjamini and Hochberg, 1995) false discovery rate of 0.05. Indeed, the lowest uncorrected *p*-value associated with any of the 1000 genes in Fig. 2A was 0.001.

Similarly, when the same synthetic data are analyzed for gene-gene correlations, one may compare the PCC values produced directly from normalized expression data with the PCC′ values produced (from unnormalized data) by BigSur. As expected, both procedures return a distribution of values with zero mean, but PCC values are more broadly distributed than PCC′. Fig. 2C shows the frequency at which various values of the correlation coefficient arise when vectors with different mean gene expression are correlated. Although significant skewing from the Fisher formula is apparent, especially at low values of gene expression, it is much greater for PCC than PCC′. Indeed, for moderately-expressed genes (e.g. 1–10 URMs per cell), only PCC′ returns values whose distribution is relatively insensitive to expression level.

### Performance on real data

To characterize the performance of BigSur on real data, we used the droplet-based sequencing data of Torre et al., 2018 (Torre et al., 2018), obtained from a clonal isolate of a human melanoma cell line grown in culture. In this data set, the number of cells is large (8,640), data were validated on a second sequencing platform as well as by single molecule FISH, and the broad distribution of sequencing depths was typical of droplet-based sequencing.

We deliberately chose a clonal cell line because tissues always contain multiple cell types, i.e., groups of cells that express large sets of genes in cell-type specific ways. In such heterogeneous samples, genes that correlate with cell type identity will necessarily be strongly and densely correlated; making the identification of correlations, in a sense, too easy—i.e., not a particularly good test of a method’s performance—and not particularly informative (one may expect to identify as correlated more or less the same genes as one finds by clustering cells and testing for differential expression between clusters).

In contrast, the use of (ostensibly) homogeneous cells forces BigSur to operate on more subtle connections—for example, those involving fluctuations in function-specific gene regulatory networks—that cell clustering and differential expression would not easily detect.

Accordingly, scRNAseq data from these cells were subjected to minimal pre-processing prior to analysis (see Methods), such that expression values were analyzed for 14,933 genes. Total UMI per cell varied greatly, ranging from 67 to 90,494, with 90% of cells containing between 666 and 9004 UMI.

**Figure 3.**
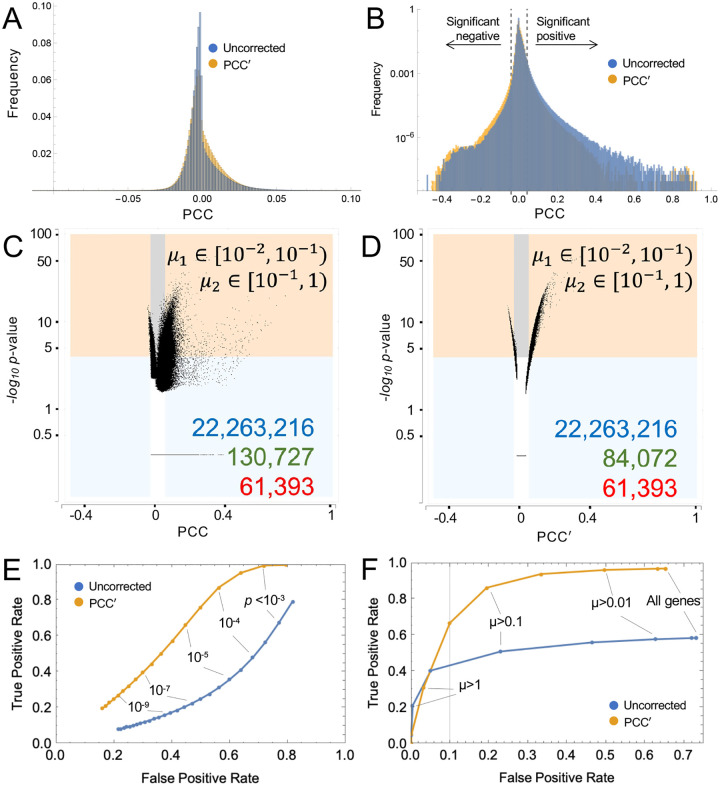
Statistical significance of pairwise gene correlations in data from a clonal cell line. **A-B.** Using scRNAseq data from a human melanoma cell line [(Torre et al., 2018); 8640 cells × 14933 genes], all pairwise values of PCC were calculated (after default normalization), as well as all pairwise values of PCC′ (without normalization). Histograms display the frequency at which different values of the correlation coefficient were encountered (in B the ordinate is logarithmically scaled to emphasize low-frequency events). PCC′ produces a more symmetric frequency distribution than PCC, reducing the excess of positive correlations and dearth of negative ones that simulations predict for PCC (Fig. 2). Dashed lines in B show that values above and below which *p*-values derived using the Fisher formula would be less than 10^−4^. **C-D.** Scatterplots showing *p*-values assigned by BigSur to pairs of genes within two representative sets of bins of mean gene expression (results for all pairwise bin combinations are given in Fig. S1–S2). The abscissa shows PCC in panel C and PCC′ in panel D. The ordinate gives the negative *log*_*10*_ of *p*-values, so that larger numbers correspond to increasing statistical significance. Orange and gray shading shows the gene pairs that BigSur judged to be significant (at FDR<0.02). Blue and orange shading shows the gene pairs that would have been judged statistically significant using the same *p*-value threshold as determined by BigSur, but calculated using the Fisher formula instead. The unshaded (white) region shows gene pairs that are not judged significant by either method; the blue region shows gene pairs judged significant according to the Fisher formula, but excluded by BigSur. Numbers in the lower right corner are the total number of possible correlations (blue), the number of statistically significant correlations according to the Fisher formula (green) and the number of statistically significant correlations according to BigSur (red). **E-F.** ROC curves assessing whether the overall performance of the Fisher formula—applied either to PCC or PCC′—can be adequately improved either by requiring a more stringent *p*-value cutoff (E), or limiting pairwise gene-correlations to those involving only genes with mean expression above a threshold level, *μ* (F).

First, we compared uncorrected PCCs for all gene-gene pairs, calculated using defaultnormalized expression data (i.e. scaled according to total expression per cell), with PCC′ values returned by BigSur (Fig. 3A–B). In panel B, frequencies are scaled logarithmically, to better display the distribution of large absolute values. BigSur associated a false discovery rate (FDR) of 2% to *p*-values less than 0.0001, at which threshold it detected 557,969 correlations, 299,135 of which were positive. For uncorrected PCCs, the same *p*-value cutoff would translate, using the Fisher formula, to |PCC| > 0.042, which is displayed as a dotted line in Fig. 3B. Using this threshold, 1,452,688 correlations would be considered significant. Comparison of histogram shapes shows that use of uncorrected PCCs particularly inflates positive correlations, especially large ones, and under-represents negative ones, which is consistent with the results obtained using simulated data (Fig. 2).

To see how the discovery of correlations varied with gene expression level, we divided genes into 6 bins of different mean expression, and separately analyzed correlations between genes in all 21 possible combinations of bins. The full data are presented in Fig. S1–S2, with two representative panels in Fig. 3C–D. Each point represents a gene-gene pair. The value on the abscissa is either the uncorrected PCC (Fig. 3C and S1) or modified corrected PCC′ (Fig. 3D and S2), and the value on the ordinate is the −*log*_*10*_ *p*-value, as determined by BigSur (i.e. the larger the number, the lower the *p*-value). Shaded territories mark those data points that were judged statistically significant (FDR<0.02) either by Big Sur (orange and gray), or according to *p*-values calculated by the Fisher formula (blue and orange; for further details see figure legend). The data confirm that the Fisher formula returns an excess of correlations, compared to Big Sur, albeit less severely for PCC′ than for PCC. Examination of the full dataset (Fig. S1–S2) shows that many more significant correlations are found among highly expressed genes; and the Fisher formula performs worst when at least one of the pairs in a correlation is a lowly-expressed gene. Indeed, as mean gene expression becomes very high (e.g. >1 UMI per cell for both genes in a pair), the distribution of *p*-values calculated by BigSur for PCC′ begins to approximate the Fisher formula reasonably well (Fig. S2), with deviation apparent only for very low *p*values (−*log*_*10*_*p* > 10). This observation validates the accuracy of the method used by BigSur to recover *p*-value distributions from the first five moments of the expected distributions of modified, corrected Pearson residuals (see Methods).

Assuming, for the sake of illustration, that the correlations judged significant by BigSur represent ground truth, we may then calculate levels of true- and false-positivity when *p*-values are calculated by feeding either PCC or PCC′ into the Fisher formula. This enabled us to ask whether applying a more stringent *p*-value cutoff, or thresholding gene expression (i.e., excluding genes below a certain expression level), might enable this simpler, formulaic approach to achieve an acceptable level of sensitivity and specificity. As shown by the receiver-operator curves in Fig. 3E, the performance of uncorrected PCCs is exceedingly poor regardless of *p*-value threshold, with the number of false positives exceeding true positives at all values. PCC′ does much better, but it is still the case that, to control FDR to <10%, one loses the ability to detect >85% of true positives.

Arbitrarily thresholding gene expression performs somewhat better (Fig. 3F). For uncorrected PCCs, one must exclude all genes with expression < 0.4 UMI/cell to control the FDR at 10%, which for this dataset means discarding 93% of all gene expression data, and in return recovering <42% of true positives. PCC′ does better here: we may recover >65% of true positives at an FDR of 10% by discarding the 91% of the genes that have lowest expression. What this suggests is that, in those cases in which one is willing to sacrifice the power to identify a substantial minority of correlations, feeding PCC′ (but not PCC) into the Fisher formula can represent an acceptable and computationally fast alternative to *p*-value identification by BigSur.

### Clustering by correlations

Although BigSur found > 500,000 statistically significant correlations in this dataset (about 0.5% of all possible pairwise correlations) the vast majority had quite small values of PCC′ (Fig. 3A–B), indicating that most correlations, while statistically significant, were weak. To obtain a measure of correlation strength that could be compared across samples of different lengths (numbers of cells), we expressed each correlation in terms of an “equivalent” PCC, which is simply the PCC value that, for continuous, normally-distributed data of the same length, would have produced the same *p*-value (by the Fisher formula). As shown in Fig. S3, only 1934 gene pairs displayed equivalent PCCs greater than 0.2 or less than −0.2.

Yet, despite the weak strength of most correlations, there are good reasons to believe most of them are biologically relevant. One of the simplest pieces of evidence comes from examining the frequency with which we detect correlations among paralogous genes and genes that encode proteins that physically interact. It is known that gene paralogs are frequently co-regulated (Ibn-Salem et al., 2017) leading us to expect paralog pairs to be enriched among bona fide correlations. It is also reasonable to expect that transcripts encoding proteins that interact will be co-expressed at least some of the time. As it happens, among the correlations identified by BigSur we observe ~15 fold enrichment for paralogs and >7.5 fold enrichment for genes encoding physically-interacting proteins [see Methods].

**Figure 4.**
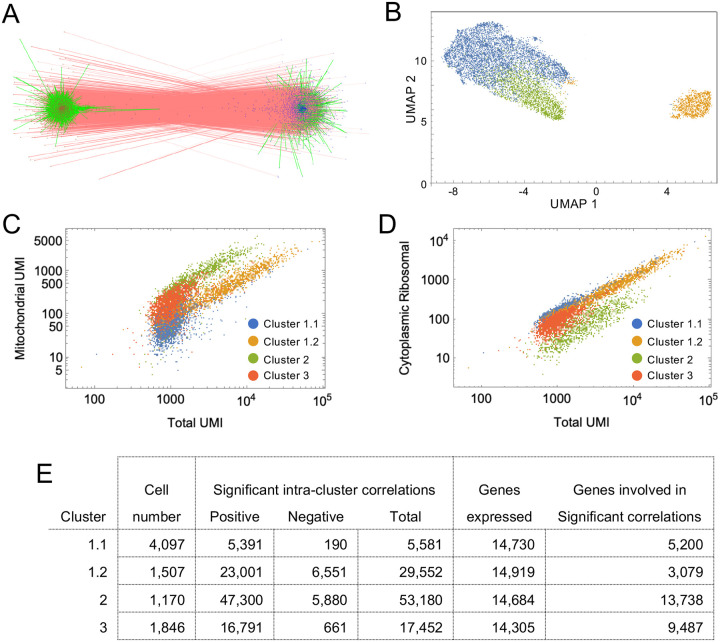
Clustering cells based on mitochondrial and ribosomal communities. **A**. Correlations among the two largest gene communities detected by BigSur are shown. Vertices are genes, and edges—green and red—represent significant positive and negative correlations, respectively. Blue vertices represent members of the mitochondrially encoded gene community and brown vertices the ribosomal protein gene community (Labels have been omitted due to the large numbers of gene involved). **B.** Using the top 75 most highly positively connected vertices in the two communities as features, cells were subjected to PCA and Leiden clustering; the three clusters recovered are displayed by UMAP. **C-D.** After a second round of clustering of Cluster 1, the four cell groups were analyzed for the distribution of expression of ribosomal protein-coding and mitochondrially-encoded genes, as a function of total UMI in each cell. The results show that the four clusters form distinct groups based on their relative abundances of ribosomal and mitochondrial genes. **E.** Results of applying BigSur to each cluster. Note the very large decrease in statistically significant correlations in any of the clusters when compared with results obtained using all of the cells together (>500,000 total correlations). This is consistent with heterogeneity in the original sample, causing large blocks of genes to be correlated with each other.

To divide the full set of correlations into potentially interpretable groups, we used a random-walk algorithm (Pons and Latapy, 2005) to identify subnetworks more highly connected internally than to other genes; we refer to these as gene communities. Most communities were of modest size, with 54 of the 56 largest containing between 5 and 213 genes each. However, the largest two contained 2223 and 1613 genes respectively, were very densely connected internally, and strongly anti-correlated with each other (Figure 4A). These factors strongly suggest that the cells assayed here are heterogenous, falling into at least two distinct groups. Interestingly, the largest of these two communities contained virtually the entire set of mitochondrially-encoded mitochondrial genes, and the second largest contained virtually all protein-coding ribosomal genes (for both cytoplasmic and mitochondrial ribosomes). Using the top 75 most-highly connected genes in each of these communities as features, we performed Leiden clustering on the modified corrected Pearson residuals of all 8,640 cells, and easily subdivided them into three clusters of 5,604, 1,170 and 1,846 cells, which we labeled as clusters 1,2 and 3, respectively (Fig. 4B; see Methods).

We then re-analyzed each cluster independently by BigSur. Surprisingly, in each cluster BigSur again found two large, strongly anti-correlating communities, one of which contained the mitochondrial-encoded genes and the other the ribosomal protein genes. This led us to further subcluster cluster 1, using the most-highly connected genes in these two communities as features, thereby subdividing it into two groups of 4,097 (cluster 1.1) and 1,507 cells (cluster 1.2).

Variable expression of mitochondrially-encoded genes is a common finding in scRNAseq. Their presence at high levels (e.g. >25% of total UMI) is usually interpreted as an indication of a “low quality” cell—potentially one in which the plasma membrane has ruptured and cytoplasm has been lost—or perhaps a cell in the process of apoptosis (Ilicic et al., 2016). Closer examination of the cell clusters identified in this dataset suggests this is likely only part of the explanation. In Fig 4C, we plot mitochondrial-encoded UMI versus total UMI for the entire set of cells, coloring them according to the four cell clusters mentioned above. Several distinct behaviors were noted. Cell cluster 2 forms a coherent group with high mitochondrial expression that is linearly proportional to total UMI. Throughout this group, the percentage of total transcripts that is mitochondrial remains in a narrow band, with mean of 34% and coefficient of variation (CV) of 0.36. Cluster 3 displays low total UMI, and a mitochondrial fraction centered around a mean of 20% (CV=0.43). Cluster 1.2 has high mitochondrial expression, but even higher total UMI, such that the mitochondrial fraction averages 9.7% (CV=0.43), while cluster 1.1 has both low total UMI and low mitochondrial UMI (average mitochondrial fraction 8.0%, CV=0.50). In Fig. 4D, we also plot total UMI derived from ribosomal protein genes against total UMI.

Of all the clusters, cluster 2 best fits the expectation for “damaged” cells: Mitochondrial and ribosomal UMI rise proportionately with total UMI (consistent with randomly varying sequencing depths), but the proportion of mitochondrial UMI is almost three times higher, the proportion of ribosomal UMI almost three times lower, and total UMI about 2.5 times lower than in cluster 1.2, the only other cluster that displays a wide range of sequencing depths. Yet it is curious that the mitochondrial proportions in cluster 2 are so narrowly distributed around a mean; one might expect variable degrees of cytoplasm leakage following cell damage to produce a smeared distribution beginning near cluster 1.2 and gradually tapering off. The absence of such behavior suggests that such cells are not merely variably damaged during preparation, but may represent a distinct, pre-existing state, perhaps associated with some form of cell stress or death (although we see no significant enrichment of gene expression associated with apoptosis (Langemeijer et al., 2016) in cluster 2).

**Figure 5.**
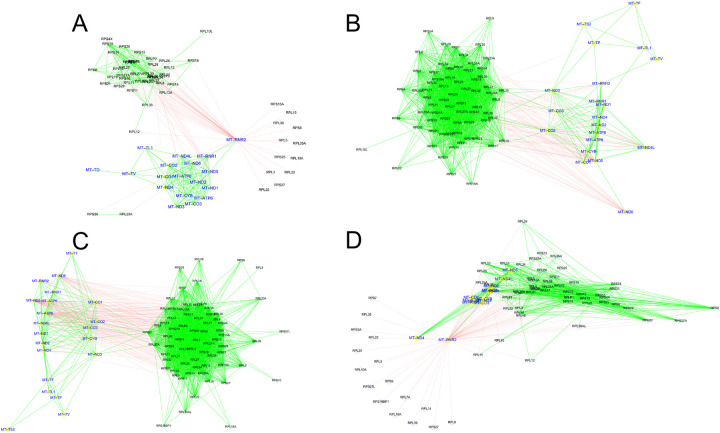
Mitochondrial and ribosomal communities are present and anti-correlate in all cell clusters. Mitochondrially-encoded and ribosomal protein gene communities identified in clusters 1.1 (**A**), 1.2 (**B**), 2 (**C**) and 3 (**D**). For ease of readability, only the mitrochondirally-encoded genes and the ribosomal protein genes are shown; labels for the former are highlighted in blue. Red links refer to negative correlations; green to positive.

Even if we remove cluster 2 from further analysis, clusters 1.1, 1.2 and 3 also differ between in each other in relative proportions of mitochondrially-encoded, ribosomal and total transcripts; however the way in which they do so is not suggestive of any simple mechanism of cell damage (and overall mitochondrial content is not in the range that would commonly lead to exclusion from downstream scRNAseq analyses). As mentioned above, using the genes in the mitochondrially-encoded and ribosomal communities as features, re-running BigSur on each of the four clusters again revealed anti-correlating communities of mitochondrially-encoded and ribosomal genes in every case (Figure 5). In fact, even after another round of subclustering of the largest cluster (cluster 1.1) using these genes as features, BigSur still identified distinct, anti-correlating mitochondrially-encoded and ribosomal communities (not shown). These data strongly suggest that, notwithstanding effects of cell damage or cell death, there exists among healthy cells continuous, anti-correlated variation in both the mitochondrially-encoded and ribosomal protein genes, suggestive of some biologically meaningful regulatory relationship (discussed further below).

### Analysis of gene communities

Figure 4E summarizes the results of using BigSur to identify significant correlations (FDR<0.02) in each of the cell clusters described above (1.1, 1.2, 2 and 3). Because statistical power to detect correlations falls with number of cells analyzed, one might expect to see the fewest correlations in the smallest clusters, but the reverse was the case. A possible explanation is that statistical power increases with the average number of UMI per cell, and some of the clusters differ markedly in total UMI (Fig. 4C). Yet cluster 1.1 has about the same number of total UMI as cluster 3, and more than twice as many cells, yet less than one third as many significant correlations. These results suggest that, in addition to there being differences related to statistical power, the results returned by BigSur may reflect differing levels of cell heterogeneity.

When a fine-grained analysis of the correlations identified in each of these clusters was carried out, many overlapping gene communities were observed (besides, as already discussed, those involving mitochondrially-encoded and ribosomal genes). Below we focus on cluster 1.2, which displayed the greatest ratio of correlations to number of genes involved (Fig. 4E), indicative of a more modest number of more densely connected networks. This pattern is consistent with low overall cell-to-cell heterogeneity, such that one might expect detected networks to be more likely to represent truly co-regulated genes rather than markers of cell types or states. Consistent with this, we found that enrichment for paralog pairs and genes whose products interact physically was much higher in this group than when all 8,640 cells had been considered together. Specifically, paralog pairs were enriched 58-fold, and links supported by protein-protein interactions 29-fold, over what would have been expected by chance. Indeed, links supported by known protein-protein interactions rose to 13.5% of all positive correlations.

Table 1 and Figures S1–S4 depict most of the large gene communities detected in cluster 1.2, which have been labeled A through M in order of decreasing size. In Figures S1–S4, genes are represented as light blue disks, except for transcription factors (yellow squares); the areas of the disks and squares reflect relative mean expression (absolute scaling is different between panels, as it was adjusted to enhance readability of each figure). Green lines between genes denote significant positive correlations (negative ones, which were not observed in these panels, would have been represented by red lines). In two cases, (Fig. S2 and S4H) graphs are shown twice, in the latter case with overlaid brown lines that highlight links supported by protein-protein interaction data.

Comparing the genes in each community with the most recent release of the MSigDB database (Mootha et al., 2003; Subramanian et al., 2005) revealed that many communities are highly enriched for functional categories, such as ribosome biogenesis, the unfolded protein response, mitochondrial function, DNA replication, mitosis, etc. (Table 1). For example, more than 52% of the genes in community A are associated with functions related to the G1 and S phases of the cell cycle, while about 77% of community B consists of genes associated with functions related to the G2 and M phases. These communities are also correlated somewhat with each other, but the community-finding algorithm easily subdivided them.

Most of the other communities show little correlation with the two cell-cycle related communities. Nearly 35% of genes in community C encode proteins involved in oxidative phosphorylation, many of which are cytoplasmically-encoded mitochondrial enzymes. More than half the genes in community F fall into two categories: cholesterol homeostasis, and melanosome biogenesis or function, and they are tightly connected to one another. Interestingly, a functional connection between melanogenesis and cholesterol homeostasis has been recognized (Schallreuter et al., 2009).

Community G consists of a particularly densely connected group of genes, 16 of which are associated with the Unfolded Protein Response, a well-studied cellular stress response that is known to be specifically and strongly activated in melanoma cells, as well as in cancers more generally (Manga and Choudhury, 2021; Rather et al., 2020; Sykes et al., 2016).

In community H we find two groups of genes tightly linked to each other: ones involved in mRNA and rRNA processing (including splicing), and additional genes (especially those encoding chaperones) involved in the unfolded protein response. Of the proteins encoded by genes in this community, more than half have known physical interactions (Fig. S4H) with others in the same community. This community is also one of the few to exhibit a substantial number of correlations with members of one of the cell cycle communities (community B).

The 20 genes in Community L include several strongly associated with glucose and lactate transport, and regulation of the glycolytic pathway, including *PDK1*, *PGK1*, *ENO2*, *SLC2A3* and *SLC16A3,* many of which are identified as targets of hypoxia. In contrast, community M (15 genes) is structured around a central core of genes annotated as interferon-inducible (*IFIT1*, *IFIT2*, *IFIT3*, *IFI44*, *HERC5*, *ISG15*, *IFIH1*), most of which are also associated with the cellular anti-viral response, plus several encoding proteins involved in apoptosis and/or growth arrest (PMAIP2, ING2, PPM1K). The fact that MAP4K4, which is upstream of Jun Kinase (JNK), correlates with several of these genes suggests a possible theme of JNK activation, potentially leading to apoptosis.

In addition to these communities, the previously-mentioned communities containing ribosomal protein genes (D) and mitochondrially-encoded genes (I) are also shown in Fig. S3 and S4, respectively. As described earlier, similar communities were found in all four cell clusters we analyzed; moreover, the mitochondrial and ribosomal genes in every one of these communities strongly anti-correlated with one another (Figure 5).

Interestingly, the mitochondrial gene community in cell cluster 1.2 also contained *PMEL*, which encodes an essential component of early melanosomes, *CD63*, encoding a cell surface tetraspanin that plays a role in trafficking PMEL to melanosomes, and *RPS6*, which encodes a ribosomal subunit (Table 1; *PMEL* was also observed in the mitochondrial gene communities in the other three cell clusters [Table S1]). Possible connections between these molecules and mitochondrial function include evidence that melanosomes and mitochondria form physical contacts (Daniele et al., 2014); that CD63 marks extracellular vesicles that mediate a secretory form of mitophagy (Tan et al., 2022); and that RPS6 has a variety of extra-ribosomal “moonlighting” functions, one of which is to control melanocyte proliferation via activation of p53 (McGowan et al., 2008).

As also shown in Table S1, the ribosomal communities from the four cell clusters all included *ALDOA*, *ATP5E*, *ATP5EP2*, *COX7C*, *CSTB*, *FTH1*, *FTL*, *GAPDH*, *H2AFZ*, *LRRC75A*-*AS1*, *NME1*, *PABPC1*, *PABPC3*, *PRDX1*, *S100A6*, *TAF1D*, *TMSB10*, *TPT1*, *TUBA1B*, *UBA52*, *UQCRH*, and *UQCRHL*, a collection of genes variously associated with glycolysis, oxidative phosphorylation, iron metabolism, nucleotide metabolism, and (not surprisingly) ribosome biogenesis. In addition, in three of the four cell clusters, *MLANA*—which encodes a gene required for *PMEL* processing and melanosome biogenesis—was also a member of the ribosomal community.

### Evaluating the performance of BigSur

**Figure 6.**
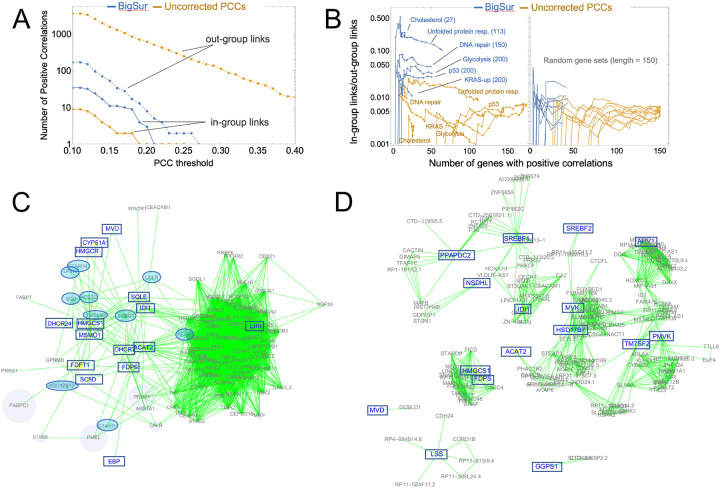
Comparing the ability of BigSur and uncorrected PCCs to identify biologically significant correlations. Using the data from cell cluster 1.2, BigSur identifed positive correlations and their p-values for all genes, converting the p-values to equivalent PCCs. In addition, the same starting data were also normalized and uncorrected PCCs obtained. A-D compare the correlations identified by the two methods. **A**. Positive correlations involving any of the genes belonging to a 27-gene panel, “Reactome Cholesterol Metabolism” were divided into “in-group” links (correlations between panel members) and “out-group” links (correlations between a panel member and another gene). With BigSur, the ratio of in-group to out-group links is about 0.2; with uncorrected PCCs it is much lower (~0.1) suggesting a much lower degree of enrichment for functionally relevant connections. **B.** The same analysis as in A was performed for five additional MSigDB panels: “Hallmark Glycolysis”, “Hallmark DNA repair”, “Hallmark p53 pathway”, “Hallmark KRAS up”, and “Hallmark Unfolded Protein Response”. Plotted are the in-group/out-group ratios over a range of PCC thresholds represented parametrically by the number of panel-members that were judged to have significant correlations. Numbers of genes in each panel are shown in parentheses. The unlabeled curves at right present results obtained using ten 150-gene sets chosen at random after first removing genes with mean expression below 0.044 (so that median expression for random genes was similar to that of the functional panel genes. **C.** The correlation community identify by BigSur for the Reactome Cholesterol Metabolism panel. Genes belonging to the panel are highlighted with boxes; known cholesterol metabolism-related genes are highlighted with rectangles. Ovals highlight genes that belong to other cholesterol-related gene sets. **D.** Correlation communities for the Reactome Cholesterol Metabolism panel identify using uncorrected PCCs.

Few instances exist in which biologists have access to actual ground truth information about gene expression correlations in single cells. Nevertheless, if we consider a set of genes involved in a common biological function, we might expect their frequency of positive correlation with each other (“in-group” links) to exceed the frequency of their positive correlation with other genes (“out-group” links), In Figure 6, we assess whether the output of BigSur indeed shows this pattern, comparing the results with ones obtained when correlations were identified using uncorrected PCCs, instead of BigSur.

Here we again consider the 1507 cells of cluster 1.2, focusing in Figure 6A on a 27-gene panel, the “Reactome Cholesterol Biosynthesis” gene set from MSigDB (*ACAT2, ARV1, CYP51A1, DHCR24, DHCR7, EBP, FDFT1, FDPS, GGPS1, HMGCR, HMGCS1, HSD17B7, IDI1, IDI2, LBR, LSS, MSMO1, MVD, MVK, NSDHL, PPAPDC2, PMVK, SC5D, SQLE, SREBF1, SREBF2, TM7SF2)*. Assigning a statistical significance threshold corresponding to an equivalent PCC of 0.117, BigSur identified positive correlations involving 15 of these genes. 35 were in-group and 170 out-group. In contrast, at the same PCC threshold, unnormalized PCCs identified positive correlations involving 26 of these genes, but only 9 correlations were internal while 3609 were external. Since it is not necessarily the case that the PCC significance threshold calculated by BigSur is appropriate to use for uncorrected PCCs, we continuously varied the threshold used for both methods, tracking the numbers of internal and external links in both cases. As Fig. 6A shows, no matter the threshold, BigSur returned a much higher ratio of in-group to out-group links.

In Fig. 6B we extend this analysis to five other function-related gene sets, as well as to synthetic gene sets composed of genes chosen at random (see Methods). The abscissa displays the number of genes with positive correlations that were ascertained over a wide range of significance thresholds from PCC=0.117 to 1. The ordinate gives the ratio of in-group to out-group links (note the logarithmic scale). For every functionally relevant gene set examined, at any significance threshold, BigSur identified far fewer out-links than uncorrected PCCs. Moreover the in-group/out-group ratios obtained with uncorrected PCCs and functionally relevant gene sets were similar to those obtained using uncorrected PCCs and random gene sets, implying the numbers of links found in both cases was at chance level. In contrast, with BigSur, the in-group/out-group ratio was much greater for functionally related gene sets than for random ones.

In Figure 6C–D, we contrast the individual correlations obtained by BigSur (panel C) with those obtained using uncorrected PCCs (panel D) for the “Reactome Cholesterol Biosynthesis” gene set, using a cutoff for uncorrected PCCs (0.28) that returned a similar number of correlated gene set members as did BigSur. With BigSur (Fig. 6C), we observed that the 15 detected members of the gene set (highlighted with blue rectangles) all formed a single community, with many internal linkages (the large densely connected mass on the righthand side represent cell cycle-related genes that happen correlate with *LDR*, as well as strongly with each other). Highlighted by blue ovals are a set of 10 additional genes that were not in the Reactome Cholesterol Biosynthesis gene set, yet on further investigation were found to be members of other cholesterol metabolism-related gene sets (in MSigDB). Thus, BigSur not only identified expected links among genes provided as input, it identified a large number of other functionally-related genes. The apparent accuracy with which BigSur did so suggests that other members of the community may also relate to cholesterol metabolism in ways not yet anticipated by current gene set databases. Indeed, the two additional genes that were most highly connected to the cholesterol-related group (Fig. 6C) were *PRDX1* (peroxiredoxin 1) and *GPNMB (glycoprotein numb)*. Peroxiredoxin protects cells against reactive oxygen compounds; it may be co-regulated with cholesterol genes either because oxidation occurs at several steps during sterol biosynthesis, or because cholesterol, once produced, needs to be protected from free-radical oxidation, to which it is prone. Numb is best known as a regulator of Notch signaling, but is in fact a clathrin adaptor protein that, besides controlling Notch internalization, is known to control internalization of the cholesterol transporter NPC1L1 (Abudoukelimu et al., 2015).

In contrast, when using uncorrected PCCs (Fig. 6D), the 16 genes of the Reactome Cholesterol Biosynthesis gene set that were found to exhibit positive correlations did not form a single community, but rather separated into 6 disconnected groups. Most groups contained links to many genes, none of which appeared, on inspection, to bear any relationship to cholesterol metabolism. These data support the view that most of the links detected using uncorrected PCCs are, in fact, false positives.

## Discussion

The above results suggest that *bona fide* communities of co-regulated genes can be identified with high specificity by carefully mining weak correlations within groups of a few thousands of relatively homogeneous cells. BigSur achieves the accuracy to do this first by correcting measures of correlation for unequal sequencing depth and the added variance contributed by gene expression noise, and subsequently by estimating an individual *p*-value for each gene pair—thereby overcoming the strong effect of gene expression distribution on the likelihood of any correlation coefficient arising by chance.

In developing BigSur, we sought to avoid normalization steps and the use of expression thresholds, and minimize user-provided parameters to the greatest extent possible. The major user input to BigSur, besides a UMI matrix, is a coefficient of variation for gene expression noise, *c*, which we argue here can be quickly estimated by fitting a plot of the modified corrected Fano factor against gene expression level (Fig. 2B). In reality, the magnitude of the noise of gene expression may be different for different genes, and for some the Poisson log-normal distribution may not be the best approximation of the noise. Although these factors likely degrade the performance of BigSur, we note that the value of *c* only significantly impacts the highly expressed genes, for which (due to low sparsity) the detection of correlations is a less challenging task.

A more subtle source of potential error comes from fact that, in calculating modified corrected Pearson residuals, the value of *μ*_*ij*_ used by BigSur is determined empirically from a finite set of cells, i.e. it is an estimator of *μ*_*ij*_. Furthermore, whereas it is an unbiased estimator when *μ* is Poisson-distributed, this is not generally the case for more skewed distributions, such as Poisson-lognormal (Lause et al., 2021). It is unknown whether these sources of error have much impact on the determination of correlation coefficient *p*-values by BigSur, and additional work will be necessary to investigate this question.

Despite these concerns, the ability to BigSur to identify gene communities that are closely related in function, as well as add new functionally-related genes into known gene sets, suggests that it already operates at a level of accuracy that can be useful to cell and tissue biologists. Particularly interesting are the questions it raises about linkages between communities—for example, why do the heavily metabolism-focused genes in community C correlate strongly with genes encoding immunomodulatory factors, such as integrin aE (*ITGAE*), macrophage migration inhibitory factor (*MIF*), galectin 1 (*LGALS1*) and galectin 3 (*LGALS3*). Should we expect metabolism and immune cell interactions to be coupled in some way, at least in melanoma?

The data in Table 1 also suggest that new cell biology may be discovered by examining those community genes labeled “not accounted for”, i.e. genes that are associated with a community but do not exhibit substantial overlap with any MSigDB dataset. For example, many genes that correlate with cell cycle communities A and B are not currently annotated as cell-cycle related. Among the strongly-coupled unfolded-protein response genes in community G, one also finds genes associated with serine biosynthesis (*PHGDH, PSAT1, SHMT2*), as well as others associated with extracellular matrix (*LAMB1, MIA*) and growth factor signaling (*GDF15, VEGFA, IL1RAP*). Indeed, each of the communities in Table 1 suggests new and unexpected forms of co-regulation of gene expression. It will be interesting to see how many of these are reproduced in other cell types—a task that can be efficiently approached by applying BigSur to the large number of already existing scRNAseq data sets.

It is instructive to note that few of the gene-gene relationships detected by BigSur could have been revealed by the typical analytical steps of cell clustering and identification of differentially expressed genes, as most of the communities identified here are not associated with sufficient total variation in gene expression to be useful drivers of cell clustering. On the other hand, clustering did play an important role here in reducing the heterogeneity of the sample to which BigSur was applied. Although essentially all the gene communities that were identified using the 1507-cell subcluster 1.2 were also observed when BigSur was applied to the entire sample of 8640 cells (not shown), clusters were more difficult to visualize and analyze thanks to a large background of cell-type (or cell-state)-specific gene expression (which generated numerous additional correlations). This experience suggests that iterative application of BigSur analysis and clustering (potentially using correlated gene communities as features) can provide a useful pipeline for identifying meaningful gene communities of manageable size.

It is interesting that one of the strongest axes of variation we detected in this study involved mitochondrially-encoded genes and genes coding for ribosomal subunits, with both communities strongly anti-correlating with each other (both before and after “damaged” cell removal and subclustering). Because the mitochondrial community consists only of mitochondrially-encoded, and not cytoplasmically-encoded, mitochondrial genes, the simplest interpretation is that this community reflects cell-to-cell variation in the number of mitochondria (or, more accurately, mitochondrial genomes). What then might explain anti-correlation between mitochondrial number and transcripts for ribosomal proteins? Given that protein synthesis requires both specialized machinery (ribosomes) and a source of energy (mitochondria), one might expect to see positive, rather than negative, correlation between the agents that orchestrate these processes. Yet this intuition is correct only in a long-time-averaged sense, and does not necessarily apply if demand for protein synthesis fluctuates. In mammalian cells, ribosomal mRNAs are long-lived, with half-lives in the 5–10 hour range (Eisen et al., 2020), whereas mitochondria can replicate on a time scale of 1–2 hours (Davis and Clayton, 1996); accordingly, one process may systematically lag the other, producing the kind of anti-correlation we observe here. While this interpretation is purely speculative, it demonstrates how the analysis of gene-gene correlations can motivate new hypotheses about transcriptome-scale regulation of cell biology.

Under the expectation that most networks of gene correlation reflect shared transcriptional regulation, we might have expected to identify upstream transcription factors more frequently in most of the networks we discovered. In some cases, this clearly did occur: For example, the transcription factors *ATF4*, *AT6*, and *XBP1* are known controllers of the unfolded protein response (community G). Transcription factors related to cell cycle progression and DNA damage-repair strongly associated with cell-cycle communities A and B. *ZKSCAN1*, a transcription factor targeted to mitochondrial (Yao et al., 2017) associates with the mitochondrially-encoded gene community. However, in many cases, transcription factors were either not observed, or those observed were not known to be connected to the processes associated with those communities. This may reflect the fact that gene regulation is often achieved through the post-translational modification of transcription factors, rather than the regulation of their mRNAs. Alternatively, it may reflect the importance of factors that act post-transcriptionally, such as miRNAs (which are not detected here), in controlling gene expression. In future, it will be important to extend BigSur to take account not only of miRNAs but also of “multi-omic” features, such as chromatin accessibility.

Although we have focused here primarily on the use of BigSur as a tool for the discovery of gene-gene correlations, it is worth pointing out that the intermediate steps in the BigSur pipeline produce useful tools for other analytical procedures, some of which were exploited here. For example, methods for feature selection for cell clustering commonly involve thresholds (e.g. expression levels) and cutoffs (e.g. numbers of features) that are arbitrary, and may not be ideal choices for every data set. Use of the modified corrected Fano factor *ϕ*′, and its associated *p*-values, can provide a less arbitrary approach to feature selection, which can outperform other methods on challenging tasks, such as finding rare cell types, or subclustering cells that differ only modestly (Dollinger et al., 2023). In addition, as we did here when dividing cells into subclusters based on the expression of ribosomal and mitochondrial genes (Fig. 4), one may also use communities of correlated genes themselves as features for clustering—in this way leveraging not just variation but co-variation to drive clustering. Finally, whereas it is common practice to cluster cells based on their normalized expression values, the matrix of modified corrected Pearson residuals that BigSur calculates almost certainly provides a more accurate starting point for clustering, as it avoids artifacts introduced by normalization, and corrects for the inflated variance associated with highly-expressed genes.

## Methods

As the first step in calculating *ϕ*′ and PCC′, we begin with “raw” (neither normalized nor log-transformed) UMI data and calculate, for each gene in each cell, a cell- and gene-specific Pearson Residual, defined according to eq. 1. To do so, we first calculate a cell- and gene-specific expected value (*μ*_*ij*_) which we obtain for each gene by averaging its values over all cells, then scaling that in each cell by to the relative proportion of total UMI that each cell contains. This is essentially the same procedure used in the simplest form of normalization, except that, rather than normalize the data matrix, we are normalizing the term for the mean in the Pearson residual.

Next, each modified Pearson residual is divided by $\sqrt{\left(1+{\text{c}}^{2}\mu_{ij}\right)}$, where *c* is a constant between 0.2 and 0.6. Ideally, *c* should be chosen individually for each gene, depending on prior knowledge of the level of gene expression stochasticity, however, in the absence of prior knowledge we typically find *c* empirically (see Fig. 2B). It should be noted that, for many scRNAseq data sets, most values of *μ*_*ij*_ will be < 1, meaning the effect of the choice of *c* on most of the data is often relatively small. To obtain *ϕ*′ for each gene, the Pearson residuals for each cell are squared, summed, and divided by *n*-1, where *n* is the number of cells.

To calculate *p*-values for *ϕ*′ any given gene, we consider the null hypothesis to be the expected number of transcripts in each cell is *μ*_*ij*_, i.e., the total number of transcripts across all the cells partitioned in proportion to the number of total UMI in each cell. As noted above, because BigSur determines the value of *μ*_*ij*_ empirically—by summing up genes UMI across all cells and multiplying by the fraction of total UMI in each cell—*μ*_*ij*_ is technically an estimator of the cell-specific expectation value for each gene and cell.

To calculate *p* we need to know how the sums of squared Pearson residuals should be distributed for any given set of *μ*_*ij*_ and *c*. As discussed above, we take *μ*_*ij*_ to have a Poisson-log-normal distribution, allowing us to calculate the moments of the distribution of squared Pearson residuals from the moments of the Poisson and log-normal distributions, and from there the moments of the distribution of sums of squared Pearson residuals. In the end we obtain, for each gene *j*, a finite set of moments (typically 4 or 5) for the distribution of *ϕ*′ that would be expected under the null hypothesis for that particular gene, given the values *μ*_1*j*_, *μ*_2*j*_, *μ*_3*j*_ … *μ*_*nj*_ and *c*. We then use Cornish-Fisher approximation of the Edgeworth expansion (Cornish and Fisher, 1938) as a reasonably computationally efficient way to approximate the *p*-value associated with any given observation of *ϕ*′, given that distribution.

The procedure for obtaining PCC′ proceeds in the same fashion, starting with the same modified corrected Pearson residuals, but now taking the dot product of the vectors of Pearson residuals for each pair of genes, and dividing by *n*-1 times the geometric mean of the *ϕ*′ values for those genes (eq. 2). Moments of the expected distributions of PCC′ are calculated analytically in exactly the manner described above, with the slight complication that the *ϕ*′ terms in the denominator are not strictly independent of the Pearson residuals in the numerator, but to a good first approximation may be treated as such, as they aggregate information across all the Pearson residuals. The Cornish-Fisher approximation is again used, as described above, to assign *p*-values to both tails of the resulting distribution. In solving the 4^th^ degree polynomial equations produced by this method (a computationally slow step), we improve speed by sacrificing accuracy specifically for those *p*-values that clearly fall below thresholds of interest.

Because the number of possible gene-gene correlations scales roughly as the square of the number of genes, the need to correct for multiple hypothesis testing is particularly acute. Given that the observations are not independent from one another, Bonferroni correction is clearly too conservative, and thus we use the Benjamini-Hochberg algorithm (Benjamini and Hochberg, 1995) for controlling the false discovery rate.

### Simulating scRNAseq data

In Figure 2 we simulate the expression of 1000 genes across 999 equivalent cells, where by equivalent we mean that, for each gene, a single “target” transcript level was chosen from a log-normal distribution, the mean of which was selected so that the logarithm of its value varied evenly across the gene set, over the range from 0.035 to 3467 transcripts per cell. The coefficient of variation of each log-normal distribution was taken to be 0.5 for all genes. Next, we generated a set of 999 scaling factors, drawn from a log-normal distribution with mean of 1 and coefficient of variation of 0.75, selected so that the logarithm of the value varied vary smoothly across the range. Finally, we generated a UMI value to each gene in each cell that was a random variate from a Poisson distribution with a mean equal to the target for that gene times the scaling factor for that cell. The result was a set of gene expression vectors of length 999, with mean values varying between 0.001 and 231.

### Analysis of melanoma cell line data

Data from droplet-based sequencing of subcloned WM989 melanoma cells (GEO accession GSE99330), which had been pre-processed to remove UMI judged not to be associated with true cells, were imported and further pre-processed in the following way: First we removed all known pseudogenes (comprehensive lists of human pseudogenes were obtained from HGNC and BioMART). Pseudogenes derived by gene duplication or retrotransposition are often highly homologous to their parent genes, creating ambiguity in the mapping of the short-read sequences used in scRNAseq. When pseudogenes were not removed from analysis, we frequently detected strong correlations between pseudogenes and parent genes that very likely represented represented the effects of ambiguous mapping, rather that true correlation. After pseudogene removal, the number of detected genes was 27,526. As both theory (Fig. 1) and experience indicated that statistically significant correlations were usually unobservable for genes with UMI in fewer than 0.001% of cells, we further eliminated genes expressed in fewer than 8 of the 8640 cells; this reduced the size of the gene set to 17,451, and the number of possible unique correlations to 152,259,975 (while not strictly necessary, this step reduces computational time by ~2.5 fold, since the number of correlations to test varies approximately quadratically with the number of genes).

### Calculating paralog pair and protein-interaction enrichment scores.

A curated list of 3,132 paralogous pairs of human genes was downloaded from (Hu et al., 2022). A list of physical human protein-protein interactions was downloaded from BioGrid (https://downloads.thebiogrid.org/File/BioGRID/Release-Archive/BIOGRID-4.4.218/BIOGRID-MV-Physical-4.4.218.tab3.zip) and supplemented with additional data from HIPPIE v2.3 (http://cbdm-01.zdv.uni-mainz.de/~mschaefer/hippie/), to produce a list of 790,008 gene pairs. To calculate enrichment, we first calculated the fraction of statistically significantly positively correlated gene pairs identified by BigSur that overlapped with either the paralog-pair or protein-protein interaction pair database. Next, we removed from the databases all gene pairs involving genes not detected in the scRNAseq data and divided the remaining number by the total number of possible gene-gene correlations (i.e. *m*(*m*-1)/2, where *m* is the number of genes in the scRNAseq data) to yield the expected frequency of paralogous or interacting pairs under the hypothesis they are randomly distributed among all possible pairwise correlations. The ratio between the observed frequency and expected frequency was considered to be the fold enrichment.

### Extracting (and pooling) gene communities

BigSur generates a matrix in which rows and columns are genes, and entries are signed equivalent PCCs—which are derived by using the inverse of the Fisher formula on the *p*-values returned by BigSur, together with the signs of the values of PCC′. Only equivalent PCCs that were judged statistically significant according to a user-supplied threshold were included, all others being set to zero. This matrix was converted to an unweighted adjacency matrix (all nonzero values replaced with 1) and the *walktrap* algorithm (with a default setting of 4 steps) was used to identify initial communities (Pons and Latapy, 2005). Because this produces communities connected by both positive and negative links, each community was then subjected to a second round of community-finding, after first setting negative links to 0, thus allowing subcommunities that negatively correlate with each other to be separated.

To identify instances in which walktrap had subdivided communities too finely, we manually examined the number of positive correlations between genes in each community and each other community, recursively merging communities in which the number of inter-community correlations was particularly large (compared with the number of possible links between communities). In addition, in rare cases in which communities returned were very large (e.g. in the thousands), we subdivided them by applying walktrap an additional time.

### Cell clustering based on correlated features

Feature selection refers to the process of identifying genes that capture important dimensions of variation on which cells may be clustered. A variety of approaches have been proposed for identifying such genes, and many work equivalently under most circumstances (with tens of thousands of genes, clustering is often a highly over-determined problem). Under challenging circumstances (e.g. when the number of true difference separating clusters is small, or the number of cells in a state is small), we have shown (Dollinger et al., 2023) that *ϕ*′ is a measure of variability at least as good as others, and because Big Sur returns both *ϕ*′ and *p*-values, one may avoid selecting too large a set of features (which can defeat clustering algorithms).

It has also been pointed out, however, that not only the statistical features of individual genes, but also their interdependencies (i.e. correlations) should ideally be used to inform clustering (Bageritz et al., 2019). We recognized that the communities identified by BigSur represent ideal sources of features, particularly if we emphasize those community members that are the most highly connected to each other. We also recognized that the modified corrected Pearson residuals generated by BigSur provide a more sensible set of vectors to use as the input to clustering than either raw or normalized UMIs (for the same reasons that *ϕ*′ and PCC′ are improvements over their unmodified, uncorrected forms). Using this approach, we routinely found that well defined clusters can often be reliably obtained using sets of highly-connected genes as small as 50–75 each. This approach was used to repetitively subcluster the scRNAseq data from WM989 melanoma cells, at each step running Big Sur and using up to 75 of the most connected genes of the clusters containing most ribosomal genes, and those containing most mitochondrially encoded genes, as features.

### Graphical display of correlations

Matrices representing statistically significant correlations were plotted using the *GraphPlot* function of Mathematica software, in which vertices were arrayed either by Spring Embedding or Spring Electrical Embedding. Edges were colored green when positive and red when negative. Vertex locations were first determined according to the graph produced after deleting negative edges, after which vertices connected only by negative edges were added in. Symbols used to represent vertices were scaled so that their areas were proportional to the mean expression level of the gene represented. Correlation strengths (the absolute values of the equivalent PCCs) are not represented on these images.

## Supplementary Material

Supplement 1 **Figure S1. Statistical significance of pairwise gene correlations in data from a clonal cell line: comparing BigSur with application of the Fisher formula to values of the uncorrected PCC.** scRNAseq data were as described in Figure 3. Data points representing pairs of genes were divided into 21 bins based on the mean expression levels of each gene, and the results for each bin were plotted as described in Fig. 3C. Orange and gray shading shows the gene pairs that BigSur judged to be significant (at FDR<0.02). Blue and orange shading shows the gene pairs that would have been judged statistically significant using the same *p*-value threshold as determined by BigSur, but calculated using the Fisher formula instead. The unshaded (white) region shows gene pairs that are not judged significant by either method; the blue region shows gene pairs judged significant according to the Fisher formula, but excluded by BigSur. Numbers in the lower right corner are the total number of possible correlations (blue), the number of statistically significant correlations according to the Fisher formula (green) and the number of statistically significant correlations according to BigSur (red).
**Figure S2. Statistical significance of pairwise gene correlations in data from a clonal cell line: comparing BigSur with application of the Fisher formula to values of the modified corrected correlation coefficient, PCC**′. scRNAseq data were as described in Figure 3. Data points representing pairs of genes were divided into 21 bins based on the mean expression levels of each gene, and the results for each bin were plotted as described in Fig. 3D. Orange and gray shading shows the gene pairs that BigSur judged to be significant (at FDR<0.02). Blue and orange shading shows the gene pairs that would have been judged statistically significant using the same *p*-value threshold as determined by BigSur, but calculated using the Fisher formula instead. The unshaded (white) region shows gene pairs that are not judged significant by either method; the blue region shows gene pairs judged significant according to the Fisher formula, but excluded by BigSur. Numbers in the lower right corner are the total number of possible correlations (blue), the number of statistically significant correlations according to the Fisher formula (green) and the number of statistically significant correlations according to BigSur (red).
**Figure S3. Distribution of equivalent PCCs.** The *p*-values obtained by BigSur for the melanoma cell line were transformed using the inverse of the Fisher formula to a set of “equivalent” PCCs. Since the Fisher formula operates on the absolute values of correlations, each calculated equivalent PCC was assigned the sign of the PCC′ value for the same gene pair. Equivalent PCCs provide a measure of correlation strength that can be compared across data sets with differing numbers of cells. They may be understood as a measure of how strongly correlated two normally distributed vectors (of any given length) would need to be to produce the observed p-value. Here, only those gene pairs judged significant by BigSur are shown. The fact that so many weakly correlated genes are statistically significant is a function of the long vector length in this experiment (>8000 cells).
**Figure S4.** Gene communities A (G1/S phase) and B (G2/M phase) from cell cluster 1.2. Green edges depict significant correlations. Transcription factor vertices are displayed as yellow boxes with gene names in blue.
**Figure S5.** Gene community C (cellular respiration, glycolysis, spliceosome, proteasome) from cell cluster 1.2. In the bottom panel gene links that correspond to known protein-protein interactions are highlighted.
**Figure S6.** Gene communities D (ribosomal protein genes), E (endosomes and stress response) and F (cholesterol metabolism, pigmentation) from cell cluster 1.2
**Figure S7.** Gene communities G (unfolded protein response), H (RNA processing) and I (mitochondrially-encoded), J (EMT, hypoxia, oxidative stress), L (hypoxia, glycolysis) and M (interferon-inducible) from cell cluster 1.2. In the second panel of H, gene links that correspond to known protein-protein interactions are highlighted.
**Table S1.** Mitochondrially-encoded and Ribosomal Protein gene communities identified in each of the four cell clusters

## Figures and Tables

**Table 1:** Gene communities identified using cell subcluster 1.2. Communities are presented in order of size. Genes were assigned after manually exploring overlaps with all MSigDB datasets (category labels often reflect the merger of redundant or semi-redundant gene sets). Genes marked “Not accounted for” failed to overlap significantly with known datasets (i.e. overlaps involved at most two genes, or accounted for less than 1% of genes in the dataset).

Community	Number of genes	Cateogory	Gene Names
A	234 genes	DNA replication, DNA repair, G1/S phase	ARL6IP6, ASF1B, ATAD2, BARD1, BLM, BRCA1, BRCA2, BRIP1, CASP8AP2, CCDC14, CCNE2, CDC25A, CDC45, CDC6, CDC7, CDCA7, CENPM, CENPQ, CENPU, CHAF1A, CHAF1B, CHEK1, CHTF18, CLSPN, COPS3, DEK, DHFR, DNA2, DONSON, DSCC1, DTL, DUT, E2F7, E2F8, EME1, ESCO2, EXO1, EZH2, FAM111B, FANCA, FANCB, FANCL, FBXO5, FEN1, GEN1, GINS1, GINS2, GINS4, GMNN, HELLS, ICMT, KIAA0101, KLHL23, LIG1, MASTL, MBD4, MCM10, MCM2, MCM3, MCM4, MCM5, MCM6, MCM7, MCM8, MNS1, MSH2, MSH6, MYB, NASP, NPAT, NUDT21, ORC1, ORC6, PARP1, PARP2, PCNA, PKMYT1, POLA1, POLD3, POLE3, POLQ, PRIM1, PRKDC, RAD18, RAD51, RAD51C, RAD54B, RBBP4, RBBP8, RBL1, RFC1, RFC2, RFC3, RFC4, RFC5, RIF1, RMI1, RPA1, RPA2, RPA3, RRM1, SKP2, SLBP, SMC1A, SRSF10, STRA13, SVIP, TFDP1, TIMELESS, TIPIN, TK1, TOPBP1, TYMS, UBE2T, UNG, USP1, USP37, WDHD1, WDR76, XRCC2, XRCC5, YEATS4, ZWINT
		Mitotic spindle, kinetochore	CENPH, CENPK, DSN1, ERCC6L, HAUS1, HAUS6, KNTC1, NCAPD3, NCAPH2, NSL1, NUP107, NUP85, TUBG1
		Nucleoside monophosphate metabolism, Tetrahydrofolate metabolic process	GART, GMPS, MTHFD1, PAICS, PPAT, PRPS2, SHMT1
		Pentose phosphate pathway	DERA, PRPS2
		*Not accounted for (38.5%)*	*ACAA2, ANKRD32, ATAD5, C19orf48, C21orf58, C3orf14, CARHSP1, CCDC109B, CCDC15, CCDC34, CCDC77, CDC5L, CDK5RAP3, CENPJ, CEP152, CEP57, CMC2, CSE1L, CTDSPL2, CTNNAL1, DNAJC9, DNMT1, ELOVL6, EMP2, EXOSC8, EXOSC9, FAM111A, FAM92A1, FARSB, FGD5-AS1, FIGNL1, FKBP5, FN3KRP, GGCT, H2AFY, HADH, HAT1, HIST1H1B, HIST1H1D, HMOX1, HNRNPAB, HNRNPF, HSPB11, IMMT, ITGA10, JPH1, KDELC2, KIF22, LMNB1, LRR1, LRRCC1, LSM8, MCMBP, MGME1, MRPL17, MRPL37, MYBL1, NAP1L4, PM20D2, PMS1, POLR3K, POP7, PPM1G, PSIP1, PSMC3IP, PTPRG-AS1, RANBP1, RBBP7, RECQL, RNASEH2A, RNASEH2B, RNF219, RRM2, SDHA, SIVA1, SLC43A3, SMCHD1, SNRNP25, SRSF2, SUPT16H, SYNE2, TEX30, TIMM21, TMEM106C, TPM4, TRIM37, VRK1, WBP11, WDR34, ZGRF1*
B	222 genes	Cell cycle G2 phase, M phase, Mitotic spindle, Cytokinesis	ANLN, ANP32B, ANP32E, ARHGAP11A, ARHGEF39, ARL6IP1, ASPM, AURKA, AURKB, BIRC5, BRD8, BUB1, BUB1B, BUB3, CASC5, CBX1, CBX5, CCNA2, CCNB1, CCNB2, CCNF, CDC20, CDC25B, CDC27, CDCA2, CDCA3, CDCA5, CDCA8, CDK1, CDKN1B, CDKN2C, CDKN3, CENPA, CENPE, CENPF, CENPN, CEP55, CEP70, CHEK2, CIT, CKAP2, CKAP2L, CKAP5, CKS1B, CKS2, CNTRL, DBF4, DCP2, DDX39A, DEPDC1, DEPDC1B, DLGAP5, DNAJB1, DTYMK, DYNLL1, ECT2, FAM64A, FAM83D, FANCD2, FOXM1, G2E3, GAS2L3, GPSM2, GTSE1, H2AFV, H2AFX, H3F3B, HAUS8, HIST1H4C, HJURP, HMG20B, HMGB2, HMGB3, HMGN2, HMMR, HN1, HP1BP3, HPS4, HSP90AA1, HSPA8, INCENP, ITGB3BP, KBTBD2, KIAA1524, KIF11, KIF14, KIF15, KIF18A, KIF20A, KIF20B, KIF23, KIF2C, KIF4A, KIF5B, KNSTRN, KPNA2, LBR, MAD2L1, MELK, MIS18BP1, MKI67, MND1, MPHOSPH9, MTF2, MZT1, NCAPD2, NCAPG, NCAPG2, NCAPH, NDC80, NEIL3, NEK2, NUCKS1, NUF2, NUMA1, NUP37, NUSAP1, OIP5, PBK, PCF11, PCNT, PIF1, PLK1, PLK4, PPP4R2, PRC1, PRR11, PSMC3, PSRC1, PTTG1, RACGAP1, RAD21, RAN, RANGAP1, RBM8A, RCC1, RCCD1, RHNO1, SASS6, SFPQ, SGOL1, SGOL2, SHCBP1, SKA2, SKA3, SMC2, SMC4, SPAG5, SPC25, SPDL1, SRSF3, STIL, STMN1, TACC3, TMPO, TOP2A, TPX2, TRIP13, TROAP, TTF2, TTK, TUBA1B, TUBA1C, TUBB2A, TUBB4B, UBE2C, UBE2S, WHSC1, XPO1, ZWILCH
		Chromatin remodeling at centromere	CENPW, MIS18A
		Other cell cycle	FANCI, FXR1, ILF2, KIAA0586, LYAR, MITF, RAD51AP1, RHEB, SRSF7
		*Not accounted for (18.5%)*	*ACTG1, AZIN1, BRIX1, BTG3, C5orf34, CACYBP, CALM2, CCDC18, CDCA4, CHAMP1, DIAPH3, DLEU2, EIF5, FBXO43, FUBP1, GGH, GPN3, GTF2A2, HES1, HIRIP3, HIST1H1E, HMGB1, HNRNPH1, IFI16, LSM5, MAGOHB, PARPBP, PHF19, PTGES3, RBBP6, RBMX, RPL39L, RTKN2, SAE1, SCLT1, SRRT, TAF5, TCOF1, UBALD2, UGDH, UTP18*
C	123 genes	Cellular respiration, citric acid cycle, mitochorndrial electron transport chain, oxidative phosphorylation, assembly of mitochondrial complexes	ATOX1, ATP5G1, ATP5G3, ATP5J, ATP5J2, ATP5L, ATP5O, ATP6V0B, ATP6V0E1, ATP6V1G1, CHCHD2, COX5A, COX6C, COX7A2, COX7B, COX7C, COX8A, CYC1, CYCS, LDHA, LDHB, MRPL20, NDUFA2, NDUFA4, NDUFAB1, NDUFB10, NDUFB9, NDUFC1, NDUFC2, NDUFS6, PHB, PHB2, PRDX3, SDHB, SLC25A5, TIMM10, TMSB4X, UQCR10, UQCRH, UQCRHL, UQCRQ, USMG5, VDAC1
		Glycolysis	ALDOA, GAPDH, LDHA, LDHB, PKM, TPI1
		Spliceosomal SNRNP complex, regulation of RNA splicing	C1QBP, EIF5A, LSM3, LSM4, NHP2L1, NPM1, SAP18, SF3B6, SNRPB, SNRPC, SNRPD1, SNRPD3, SNRPE, SNRPF, SNRPG
		Proteasome pathway	H2AFZ, POMP, PSMA7, PSMB3, PSMB4, PSMB6, PSMB7, UBB
		*Not accounted for (43.1%)*	*ACTB, AHCY, ANAPC11, APRT, ARHGDIA, BTF3, C20orf27, CALM1, CCT3, CCT5, CDKN2A, CFL1, COA4, COMMD4, CSTB, CTSC, ENO1, FAM162A, FOSL1, FTH1, GSTP1, HINT1, HSBP1, HSPB1, HSPE1, ITGAE, LAMTOR5, LGALS1, LGALS3, MIF, NME1, PFN1, POLR2L, PPDPF, PPIA, PTMA, RBX1, RNASEH2C, S100A10, S100A11, SKP1, SLIRP, SOD1, SSBP1, SUB1, TCEB2, THOC7, TMEM167A, TRAPPC1, TRMT112, TXN, TXNDC17, YWHAZ*
D	112 genes	Ribosomal proteins	FAU, RPL10, RPL10A, RPL11, RPL12, RPL13, RPL13A, RPL14, RPL15, RPL18, RPL18A, RPL19, RPL22, RPL23, RPL23A, RPL24, RPL26, RPL27, RPL27A, RPL28, RPL29, RPL3, RPL30, RPL31, RPL32, RPL34, RPL35, RPL35A, RPL36, RPL37, RPL37A, RPL38, RPL39, RPL4, RPL5, RPL6, RPL7A, RPL8, RPL9, RPLP0, RPLP1, RPLP2, RPS 10, RPS11, RPS12, RPS13, RPS 14, RPS15, RPS15A, RPS16, RPS17, RPS 18, RPS19, RPS2, RPS20, RPS21, RPS23, RPS24, RPS25, RPS27, RPS27A, RPS28, RPS29, RPS3, RPS3A, RPS4X, RPS5, RPS6, RPS7, RPS8, RPS9, RPSA, UBA52
		Other translation genes	EEF1A1, EEF1B2, EEF1D, EEF2, EIF3F, EIF3H, EIF3K, GNB2L1, NACA
		*Not accounted for (26.8%)*	*ATP5G2, C12orf57, C1orf43, COMMD6, COX17, COX4I1, COX5B, DANCR, EPB41L4A-AS1, GAS5, HNRNPA1, LRRC75A-AS1, METTL12, MYEOV2, NAP1L1, OST4, PABPC3, PRDX5, PRSS23, RP11–669N7.2, SEC11A, SNHG5, SNHG6, SNHG8, TBCA, TMEM258, TOMM7, TPT1, UQCRB, ZFAS1*
E	82 genes	Endosome, endolysosome, lysosome, vacuole	AGA, AP2S1, ATP6V0D2, CD63, CTSA, CTSB, CTSH, CTSK, FTL, LRPAP1, LUM, NPC2, PSAP
		Environmental stress response, apoptotic signaling in response to hypoxia, response to hydroperoxide	ARPC1B, ATP5I, GPX1, MT1X, MT2A, NGFRAP1, OAZ1, PLAUR, PRDX4, TMBIM6
		Regulation of peptidase activity in apoptosis	BIRC7, CTSH, NGFRAP1, RPS27L
		*Not accounted for (70.7%o)*	*APOC1, APOD, ATP5E, ATP5EP2, BHLHE40, CD151, CD320, CD59, CKLF, CLEC11A, COX14, CST3, CYTL1, DSTN, EIF3G, ERP29, FKBP1A, FXYD3, HPGD, LGALS3BP, LPXN, MGST3, MIA, MPG, NDUFA13, PIGT, PIK3C3, PLA2G16, PON2, RAMP1, RCN3, RNF157, S100A1, S100A13, S100A6, SDF4, SEC61G, SEMA3B, SERF2, SH3BGRL3, SHFM1, SNRPD2, SPCS1, SSR4, TIMP1, TM4SF1, TMED4, TMEM147, TMEM59, TMSB10, TNFRSF12A, TPD52L1, UBL5, VGF, VKORC1, WBP5, ZNHIT1*
F	76 genes	Cholesterol, isoprenoid metabolism	ACAT2, ACSS2, C14orf1, CD9, CYP51A1, DBI, DHCR24, DHCR7, FDFT1, FDPS, HMGCR, HMGCS1, IDI1, INSIG1, LDLR, LPIN1, MDH1, MSMO1, MVD, RLBP1, SC5D, SCD, SQLE, STARD4, SUMO2, TMEM97, YY1
		Pigmentation, melanosomes	CAPG, CTSD, DCT, DHCR7, FDFT1, MLANA, MLPH, PMEL, PRDX1, RAB27A, SYTL2
		*Not accounted for (52.6%)*	*AKR1A1, ATP6AP1, B2M, BCAR3, BHLHE41, CDK2, CHCHD6, EMP1, GJB1, GRN, GSTO1, GYPC, HIBCH, HIST1H2AC, LHFPL3-AS1, LINC01531, METTL9, NDUFB6, PDZRN3, PEBP1, PIK3R3, POLR2F, PRKD3, PTTG1IP, QPCT, RP4–718J7.4, SCML4, SEPT4, SEPT6, SPAG9, SPTY2D1, STK32A, SULT1C2, TIMM50, TRIM2, TRIM63, TTYH2, UBR5, UCN2, ZNF534*
G	75 genes	Unfolded protein response, ER stress	ARF4, ASNS, ATF4, ATF6, COPB1, CRELD2, DDIT4, DNAJB11, DNAJC3, EIF4EBP1, HERPUD1, HYOU1, KDELR2, LAMB1, MANF, MIA3, MTHFD2, P4HB, PDIA3, PDIA6, PPAPDC1B, PPIB, PPP1R15A, PSAT1, SDF2L1, SEC31A, SERP1, TRIB3, VEGFA, VIMP, WARS, XBP1, XPOT
		Other ER	NUCB2, RPN1, SEC11C, SEC63, TMED9
		tRNA synthetases	EPRS, GARS, SARS, WARS, YARS
		Serine/glycine biosynthesis	PHGDH, PSAT1, SHMT2
		*Not accounted for (41.3%)*	*ACBD3, ANKRD11, ARFGAP3, ATP1A1, BCAT1, BEST1, CDK2AP2, COPB2, CTC-425F1.4, EIF1, FAM129A, FKBP2, GDF15, GHITM, IFRD1, IL1RAP, MCF2L, MTDH, NUPR1, PYGB, RCAN1, SELK, SLC39A14, SLC3A2, SLFN5, SPEN, SSR3, TCEA1, TES, TMF1, TRAM1*
H	65 genes	Spliceosome, mRNA and rRNA processing, RNP complex biogenesis	DDX1, DDX21, DKC1, DNAJC8, EIF3A, EIF3B, EIF4A3, EIF4G1, FUS, HNRNPA2B1, HNRNPA3, HNRNPC, HNRNPD, HNRNPH3, HNRNPM, HNRNPR, HSP90AB1, LSM6, LUC7L3, NCL, NOLC1, NOM1, NOP56, NOP58, PA2G4, PNO1, TSR1, WDR3, WDR43
		Unfolded protein response, heat shock protein binding	CCT4, CCT6A, DNAJA1, DNAJC2, FKBP4, HSPA4, HSPD1, HSPH1, NUDC, STIP1, TCP1
		*Not accounted for (38.5%)*	*ARCN1, ARPC2, BAZ1B, BZW1, CBX3, CFAP97, DNTTIP2, EIF5B, GSPT1, HNRNPU, KIAA0020, M6PR, MATR3, MYOIO, SEPT7, SERBP1, SSB, TOP1, TPM3, TPRKB, UCHL5, WDFY1, XRCC6, ZC3H15, ZNF326*
I	63 genes	Mitochondrially-encoded genes	MT-ATP6, MT-ATP8, MT-CO1, MT-CO2, MT-CO3, MT-CYB, MT-ND1, MT-ND2, MT-ND3, MT-ND4, MT-ND4L, MT-ND5, MT-ND6
		Fibronectin and Integrin binding	FN1, GPNMB, IGFBP5, ITGA4, MFGE8, NOV
		*Not accounted for (69.8%)*	*ADCY1, AKAP9, APP, ARID4B, BPTF, CALD1, CBLB, CD46, CDC42BPA, CEP350, CHML, EIF4G3, GOLGA8B, GOLGB1, ITM2C, KMT2C, LPCAT2, MT-RNR1, MT-RNR2, NEDD4L, PABPC1, PEG10, PLCB4, PLEKHA4, PRRX1, PTPRG, PTPRM, RB1CC1, RND3, RYBP, SERPINE2, SLC5A3, SLK, SORBS2, SPTBN1, TDRD3, TMX4, TPR, U2SURP, UBE3A, WWTR1, ZC3H11A, ZKSCAN1, ZNF704*
J	40 genes	Epithelial-mesenchymal transition	CD44, DAB2, DST, FSTL1, PMP22, SAT1, SPARC, VIM
		Hypoxia	BTG1, MXI1, P4HA1, PLEKHA2, RNF19A, SAT1, VIM
		Oxidative stress	NQO1, TXNRD1
		Pigmentation	AP1S2, EDNRB, RAB17, SLC24A5
		*Not accounted for 52.5%*	*ANKS1A, BCAN, BOD1L1, C21orf91, CHL1, DDX17, GABPB1-AS1, GCC2, GTF2I, MAGED2, MSN, PDE3B, PMP2, S100B, SDCBP, SQSTM1, SWAP70, TSPYL2, UBL3, ZMYND8, ZNF106*
K	21 genes	Proteasome	PSMB1, PSMC4, PSMD6
		*Not accounted for (85.7%o)*	*ATP6V1E1, BLVRA, CAST, FRMD4B, LINC00152, LINC00520, LINC01291, MCTS1, MIR4435–1HG, PPY, RTCB, RTN4, SLAMF7, ST13, TCEB1, TM4SF19, UFD1L, UPP1*
L	20 genes	Hypoxia, Hif1 targets, Glycolysis	ADM, ANKZF1, BNIP3, BNIP3L, ENO2, PDK1, PGK1, SLC2A3
		Pyruvate/lactate transmembrane transport	SLC16A3
		Cysteine type peptidase inhbitors	CST1, CST4
		*Not accounted for (45%)*	*CCL28, H1F0, HIST2H2AC, NDUFB4, NRN1, RGS1, ROMO1, SLAMF9, TTC3*
M	15 genes	Interferon-response; anti-viral response	CEACAM1, HERC5, IFI44, IFIH1, IFIT1, IFIT2, IFIT3, ISG15, PMAIP1, PPM1K
		*Not accounted for (33.3%)*	*CDC42EP3, ING2, MAP4K4, MCAM, SMARCA5*
N	12 genes	EGF/FGF/TNF signaling	CTGF, EGR1, IER2, NR4A1, PDE4B, PHLDA1, PLK2, SPRY2, TSC22D1
		*Not accounted for (20 %)*	*EPS8, ETV3, USP53*
O	11 genes	Apical junctions/tight junctions	MAGI1, MAGI3, PAK2
		*Not accounted for (72.7%o)*	*CCNT2, DHX38, ELMOD2, PEX1, PHKB, RANBP10, RPIA, VMP1}*
P	11 genes	Hypopigmentation	LYST, SOX10
		*Not accounted for (81.8%)*	*BROX, CCDC150, CCNL2, HMCN1, KIAA1456, PAXBP1, PRMT7, SLC30A9, SRSF11*
