# Supplementary material for: Leveraging gene correlations in single cell transcriptomic data
Source: bioRxiv. 2023 Mar 15:2023.03.14.532643. Preprint. [Version 1] doi: 10.1101/2023.03.14.532643 (PMC10055147; doi:10.1101/2023.03.14.532643)
Supplement: Supplement 1 — Figure S1. Statistical significance of pairwise gene correlations in data from a clonal cell line: comparing BigSur with application of the Fisher formula to values of the uncorrected PCC. scRNAseq data were as described in Figure 3. Data points representing pairs of genes were divided into 21 bins based on the mean expression levels of each gene, and the results for each bin were plotted as described in Fig. 3C. Orange and gray shading shows the gene pairs that BigSur judged to be significant (at FDR<0.02). Blue and orange shading shows the gene pairs that would have been judged statistically significant using the same p-value threshold as determined by BigSur, but calculated using the Fisher formula instead. The unshaded (white) region shows gene pairs that are not judged significant by either method; the blue region shows gene pairs judged significant according to the Fisher formula, but excluded by BigSur. Numbers in the lower right corner are the total number of possible correlations (blue), the number of statistically significant correlations according to the Fisher formula (green) and the number of statistically significant correlations according to BigSur (red). Figure S2. Statistical significance of pairwise gene correlations in data from a clonal cell line: comparing BigSur with application of the Fisher formula to values of the modified corrected correlation coefficient, PCC′. scRNAseq data were as described in Figure 3. Data points representing pairs of genes were divided into 21 bins based on the mean expression levels of each gene, and the results for each bin were plotted as described in Fig. 3D. Orange and gray shading shows the gene pairs that BigSur judged to be significant (at FDR<0.02). Blue and orange shading shows the gene pairs that would have been judged statistically significant using the same p-value threshold as determined by BigSur, but calculated using the Fisher formula instead. The unshaded (white) region shows gene pairs that are not [file media-1.pdf]

Table S1. Mitochondrially-encoded and Ribosomal Protein gene communities identified in each of the four cell clusters

Cell Cluster	Community	Number of genes	Gene Names
1.1	mitochondrially-encoded	56	A2M-AS1, AC144449.1, ANAPC2, AP001350.4, CCDC13-AS1, CCDC17, CD63, CRYBB2, CTB-50L17.8, CTC-425O23.5, DNAH6, FBXL8, GPNMB, H1F0, HSP90AA1, IZUMO4, LAMB1, LINC00896, MRPL44, MT-ATP6, MT-ATP8, MT-CO1, MT-CO2, MT-CO3, MT-CYB, MT-ND1, MT-ND2, MT-ND3, MT-ND4, MT-ND4L, MT-ND5, MT-ND6, MT-RNR1, MXI1, MYC, NFKBIZ, NME5, NUMA1, PMEL, RP11-119J18.1, RP11-178C3.2, RP11-206L10.9, RP11-327E2.5, RP11-486M3.3, RP11-54O7.18, RP11-600F24.7, RP11-658F2.8, RPS6, SLC3A2, SRRM2, STARD4-AS1, TMEM102, TRIB2, UBAP1L, VEPH1, ZNF256
1.2	mitochondrially-encoded	63	ADCY1, AKAP9, APP, ARID4B, BPTF, CALD1, CBLB, CD46, CDC42BPA, CEP350, CHML, EIF4G3, FN1, GOLGA8B, GOLGB1, GPNMB, IGFBP5, ITGA4, ITM2C, KMT2C, LPCAT2, MFGE8, MT-ATP6, MT-ATP8, MT-CO1, MT-CO2, MT-CO3, MT-CYB, MT-ND1, MT-ND2, MT-ND3, MT-ND4, MT-ND4L, MT-ND5, MT-ND6, MT-RNR1, MT-RNR2, NEDD4L, NOV, PABPC1, PEG10, PLCB4, PLEKHA4, PRRX1, PTPRG, PTPRM, RB1CC1, RND3, RYBP, SERPINE2, SLC5A3, SLK, SORBS2, SPTBN1, TDRD3, TMX4, TPR, U2SURP, UBE3A, WWTR1, ZC3H11A, ZKSCAN1, ZNF704
2	mitochondrially-encoded	32	ABCC11, AC110056.1, AC144652.1, COX6A1, CTSS, CXCL8, IGSF8, MGST3, MT-ATP6, MT-ATP8, MT-CO1, MT-CO2, MT-CO3, MT-CYB, MT-ND1, MT-ND2, MT-ND3, MT-ND4, MT-ND4L, MT-ND5, MT-ND6, MT-RNR1, MT-RNR2, MT-TF, MT-TL1, MT-TP, MT-TT, MT-TV, NDUFB9, PMEL, RP4-740C4.7, UBTD1
3	mitochondrially-encoded	19	CEACAM5, CFAP20, MT-ATP6, MT-ATP8, MT-CO1, MT-CO2, MT-CO3, MT-CYB, MT-ND1, MT-ND2, MT-ND3, MT-ND4, MT-ND4L, MT-ND5, NDUFB9, PAIP2B, PMEL, RP11-626G11.5, SUCLA2-AS1
1.1	ribosomal protein genes	50	COX7C, RPL11, RPL12, RPL13, RPL18, RPL19, RPL24, RPL27A, RPL28, RPL30, RPL31, RPL34, RPL37, RPL37A, RPL38, RPL8, RPLP0, RPLP1, RPLP2, RPS11, RPS12, RPS13, RPS14, RPS16, RPS19, RPS20, RPS21, RPS23, RPS27A, RPS29, RPS3, RPS5, RPS8, TPT1, UBA52, LINC00957, RPS17, CCDC78, FTH1, RP11-221H10.2, RPL10, RPL10L, RPL26, RPL27, RPS15, RPS15A, RPS18, RPS2, RP11-186N15.3, RPL13A
1.2	ribosomal protein genes	112	ATP5G2, C12orf57, C1orf43, COMMD6, COX17, COX4I1, COX5B, DANCR, EEF1A1, EEF1B2, EEF1D, EEF2, EIF3F, EIF3H, EIF3K, EPB41L4A-AS1, FAU, GAS5, GNB2L1, HNRNPA1, LRRC75A-AS1, METTL12, MYEOV2, NACA, NAP1L1, OST4, PABPC3, PRDX5, PRSS23, RP11-669N7.2, RPL10, RPL10A, RPL11, RPL12, RPL13, RPL13A, RPL14, RPL15, RPL18, RPL18A, RPL19, RPL22, RPL23, RPL23A, RPL24, RPL26, RPL27, RPL27A, RPL28, RPL29, RPL3, RPL30, RPL31, RPL32, RPL34, RPL35, RPL35A, RPL36, RPL37, RPL37A, RPL38, RPL39, RPL4, RPL5, RPL6, RPL7A, RPL8, RPL9, RPLP0, RPLP1, RPLP2, RPS10, RPS11, RPS12, RPS13, RPS14, RPS15, RPS15A, RPS16, RPS17, RPS18, RPS19, RPS2, RPS20, RPS21, RPS23, RPS24, RPS25, RPS27, RPS27A, RPS28, RPS29, RPS3, RPS3A, RPS4X, RPS5, RPS6, RPS7, RPS8, RPS9, RPSA, SEC11A, SNHG5, SNHG6, SNHG8, TBCA, TMEM258, TOMM7, TPT1, UBA52, UQCRB, ZFAS1

2	ribosomal protein genes	502	ABHD1, AC006077.3, AC006942.4, AC007131.2, AC009784.3, ACN9, ACTB, ACTC1, ACTG1, ADIPOR1, AKR1B1, AL161626.1, ALDOA, ANP32A, ANP32B, APOC1, APOD, APRT, ARF1, ARPC1A, ARPC1B, ARPC2, ARPC3, ATOH8, ATOX1, ATP5E, ATP5EP2, ATP5G1, ATP5G2, ATP5G3, ATP5I, ATP5J, ATP5J2, ATP5L, ATP5O, ATP6V0B, ATP6V1E1, ATP6V1F, ATP6V1G1, AURKAIP1, B2M, BACE1, BBX, BCAS3, BCL3, BOLA3, BTF3, BUD31, BZW2, C12orf57, C14orf2, C16orf95, C17orf89, C19orf10, C19orf53, C1orf21, C1orf43, C1orf61, C1QBP, C20orf27, C7orf57, C9orf116, CALM1, CALM2, CAPG, CAPZB, CBX3, CCDC58, CCNI, CD63, CD93, CDKN2A, CFHR1, CFL1, CH17-472G23.2, CHCHD2, CHCHD3, CHCHD6, CHMP2A, CKS2, CNBP, COA4, COX17, COX4I1, COX5A, COX5B, COX6C, COX7A2, COX7B, COX7C, COX8A, CPA2, CRHBP, CST3, CSTB, CTB-131K11.1, CTC-428G20.6, CTC-527H23.3, CTD-2313J17.5, CTD-2349P21.11, CTD-2369P2.12, CTD-2561B21.11, CTD-2623N2.11, CYCS, DBI, DCXR, DSTN, DTX1, DUT, DYNLL1, DYNLRB1, ECHS1, EDF1, EEF1A1, EEF1B2, EIF1, EIF2S2, EIF3G, EIF3H, EIF3I, EIF4EBP1, EIF5A, ELF4, EMR1, ENO1, ENY2, ERH, ESD, F8A1, FAM213B, FAU, FBL, FDFT1, FIS1, FKBP1A, FLJ43681, FTH1, FTL, FTMT, FXYD5, GADD45GIP1, GALNT18, GAPDH, GATA6, GLO1, GNAS, GNB2L1, GNG12, GPX1, GPX4, GSTO1, GSTP1, GYPC, H2AFZ, H3F3B, HBB, HEXA-AS1, HINT1, HIST1H4C, HMGA1, HMGB1, HMGN2, HNRNPC, HOXC-AS1, HSBP1, HSPB1, HSPE1, KDELR1, KIAA0101, LAGE3, LAMTOR1, LAMTOR4, LAMTOR5, LAPTM4B, LCMT1-AS1, LDHA, LDHB, LGALS1, LGALS3, LINC00087, LINC00324, LINC00493, LKAAEAR1, LLPH, LRRC75A-AS1, LSM4, LSM7, MAD2L1, MAP1LC3B, MATN3, MDH1, MDH2, MFSD2B, MIF, MIR497HG, MIR7111, MITF, MKKS, MLANA, MPC2, MPG, MRFAP1, MRPL12, MRPL13, MRPL33, MRPL48, MRPL51, MRPS28, MT1G, MT2A, MT4, MTPN, MYEOV2, MYL6, MZT2B, NACA, NAP1L1, NDUFA1, NDUFA11, NDUFA13, NDUFA4, NDUFA6, NDUFAB1, NDUFAF3, NDUFB11, NDUFB2, NDUFB3, NDUFB7, NDUFC1, NDUFC2, NDUFS5, NDUFS6, NEDD8, NGFRAP1, NHP2, NHP2L1, NIM1K, NME1, NME5, NPC2, NPM1, NSA2, NUTF2, OAS2, OAZ1, OLA1, OST4, PA2G4, PABPC1, PABPC3, PAICS, PARK7, PCDH15, PCYT1B, PDAP1, PDCD5, PEBP1, PEG10, PEX11G, PFDN2, PFDN4, PFN1, PHB, PHB2, PIR, PKM, PMP22, POLR2F, POLR2I, POLR2J, POLR2L, POT1-AS1, PPDPF, PPIA, PPIB, PRDX1, PRDX2, PRDX6, PRELID1, PRKAR1A, PRRG2, PSAT1, PSMA7, PSMB4, PSMB6, PSMF1, PTMA, PTMS, PTPRR, PYCARD, PYCR1, RAB38, RAB7A, RALY, RAN, RBX1, REXO2, RGS10, RHOA, RN7SL2, ROMO1, RP11-1100L3.8, RP11-114H23.2, RP11-147C23.1, RP11-161D15.1, RP11-162A12.2, RP11-20E24.1, RP11-229E13.4, RP11-248J18.2, RP11-255C15.3, RP11-283G6.5, RP11-293A21.1, RP11-327E2.5, RP11-338I21.1, RP11-350G8.5, RP11-360O19.4, RP11-384P7.7, RP11-424N24.2, RP11-435D7.3, RP11-46H11.12, RP11-474I16.8, RP11-651L5.3, RP11-669N7.2, RP11-672L10.6, RP11-675F6.4, RP11-6O2.4, RP11-755B10.3, RP11-807H7.2, RP11-831H9.11, RP11-872D17.4, RP13-
3	ribosomal protein genes	118	ACTG1, ALDOA, ATOX1, ATP5E, ATP5EP2, ATP5G1, C14orf2, CD63, COX4I1, COX8A, CSTB, CTD-2165H16.4, DBI, DUT, ENO1, FAM132B, FAU, FTH1, FTL, GAPDH, GNB2L1, H2AFZ, HIST1H4C, HOXB5, LDHB, LGALS1, LGALS3, LRRC75A-AS1, MLANA, MT2A, NDUFA4, NDUFC2, NME1, PCDHB4, PFN1, POLR2J, PRDX1, PSMA7, PTMA, ROMO1, RP11-331F4.5, RP11-350N15.5, RP11-666A8.8, RP11-84A14.4, RP13-941N14.1, RPL10, RPL11, RPL12, RPL13, RPL13A, RPL14, RPL15, RPL18, RPL19, RPL23, RPL23A, RPL24, RPL26, RPL27, RPL27A, RPL28, RPL30, RPL31, RPL32, RPL34, RPL35, RPL35A, RPL37, RPL37A, RPL38, RPL4, RPL5, RPL8, RPLP0, RPLP1, RPLP2, RPS11, RPS12, RPS13, RPS14, RPS15, RPS15A, RPS16, RPS17, RPS18, RPS19, RPS2, RPS20, RPS21, RPS23, RPS24, RPS25, RPS27A, RPS29, RPS3, RPS4X, RPS5, RPS6, RPS8, RSAD2, S100A6, SERF2, SHFM1, SLIRP, SNHG5, SNHG6, SOD1, STMN1, TMSB10, TOMM7, TPI1, TPT1, TXN, UBA52, UBB, UQCRB, UQCRQ, ZC3H12B

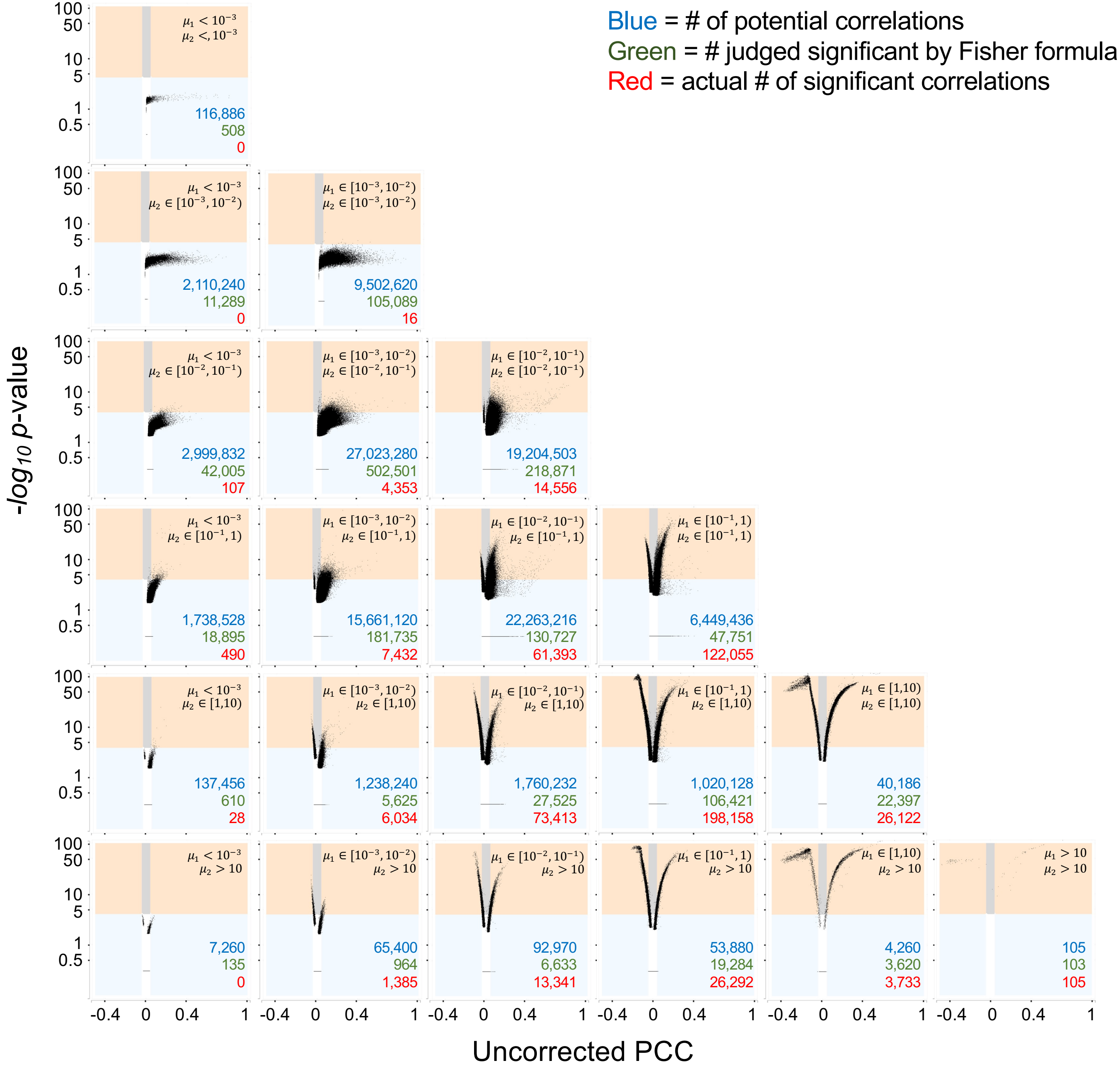

Blue = # of potential correlations
Green = # judged significant by Fisher formula
Red = actual # of significant correlations
-log10 p-value
Uncorrected PCC
$\mu_1 < 10^{-3}$, $\mu_2 < 10^{-3}$: 116,886; 508; 0
$\mu_1 < 10^{-3}$, $\mu_2 \in [10^{-3}, 10^{-2})$: 2,110,240; 11,289; 0
$\mu_1 \in [10^{-3}, 10^{-2})$, $\mu_2 \in [10^{-3}, 10^{-2})$: 9,502,620; 105,089; 16
$\mu_1 < 10^{-3}$, $\mu_2 \in [10^{-2}, 10^{-1})$: 2,999,832; 42,005; 107
$\mu_1 \in [10^{-3}, 10^{-2})$, $\mu_2 \in [10^{-2}, 10^{-1})$: 27,023,280; 502,501; 4,353
$\mu_1 \in [10^{-2}, 10^{-1})$, $\mu_2 \in [10^{-2}, 10^{-1})$: 19,204,503; 218,871; 14,556
$\mu_1 < 10^{-3}$, $\mu_2 \in [10^{-1}, 1)$: 1,738,528; 18,895; 490
$\mu_1 \in [10^{-3}, 10^{-2})$, $\mu_2 \in [10^{-1}, 1)$: 15,661,120; 181,735; 7,432
$\mu_1 \in [10^{-2}, 10^{-1})$, $\mu_2 \in [10^{-1}, 1)$: 22,263,216; 130,727; 61,393
$\mu_1 \in [10^{-1}, 1)$, $\mu_2 \in [10^{-1}, 1)$: 6,449,436; 47,751; 122,055
$\mu_1 < 10^{-3}$, $\mu_2 \in [1,10)$: 137,456; 610; 28
$\mu_1 \in [10^{-3}, 10^{-2})$, $\mu_2 \in [1,10)$: 1,238,240; 5,625; 6,034
$\mu_1 \in [10^{-2}, 10^{-1})$, $\mu_2 \in [1,10)$: 1,760,232; 27,525; 73,413
$\mu_1 \in [10^{-1}, 1)$, $\mu_2 \in [1,10)$: 1,020,128; 106,421; 198,158
$\mu_1 \in [1,10)$, $\mu_2 \in [1,10)$: 40,186; 22,397; 26,122
$\mu_1 < 10^{-3}$, $\mu_2 > 10$: 7,260; 135; 0
$\mu_1 \in [10^{-3}, 10^{-2})$, $\mu_2 > 10$: 65,400; 964; 1,385
$\mu_1 \in [10^{-2}, 10^{-1})$, $\mu_2 > 10$: 92,970; 6,633; 13,341
$\mu_1 \in [10^{-1}, 1)$, $\mu_2 > 10$: 53,880; 19,284; 26,292
$\mu_1 \in [1,10)$, $\mu_2 > 10$: 4,260; 3,620; 3,733
$\mu_1 > 10$, $\mu_2 > 10$: 105; 103; 105

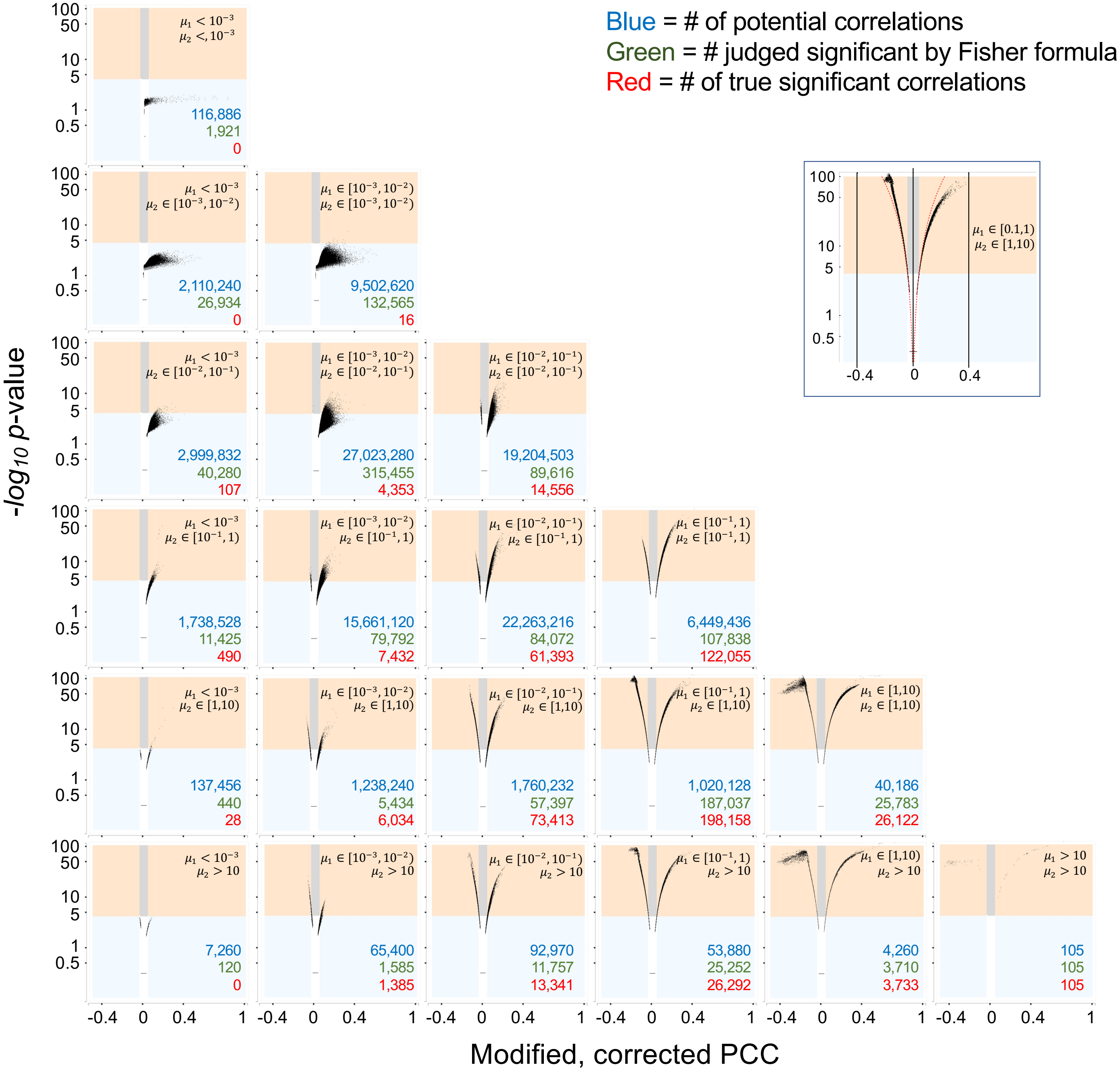

Blue = # of potential correlations
Green = # judged significant by Fisher formula
Red = # of true significant correlations
-log10 p-value
Modified, corrected PCC
100
50
10
5
1
0.5
-0.4
0
0.4
1
$\mu_1 < 10^{-3}$ $\mu_2 <, 10^{-3}$
116,886
1,921
0
$\mu_1 < 10^{-3}$ $\mu_2 \in [10^{-3}, 10^{-2})$
2,110,240
26,934
0
$\mu_1 \in [10^{-3}, 10^{-2})$ $\mu_2 \in [10^{-3}, 10^{-2})$
9,502,620
132,565
16
$\mu_1 < 10^{-3}$ $\mu_2 \in [10^{-2}, 10^{-1})$
2,999,832
40,280
107
$\mu_1 \in [10^{-3}, 10^{-2})$ $\mu_2 \in [10^{-2}, 10^{-1})$
27,023,280
315,455
4,353
$\mu_1 \in [10^{-2}, 10^{-1})$ $\mu_2 \in [10^{-2}, 10^{-1})$
19,204,503
89,616
14,556
$\mu_1 < 10^{-3}$ $\mu_2 \in [10^{-1}, 1)$
1,738,528
11,425
490
$\mu_1 \in [10^{-3}, 10^{-2})$ $\mu_2 \in [10^{-1}, 1)$
15,661,120
79,792
7,432
$\mu_1 \in [10^{-2}, 10^{-1})$ $\mu_2 \in [10^{-1}, 1)$
22,263,216
84,072
61,393
$\mu_1 \in [10^{-1}, 1)$ $\mu_2 \in [10^{-1}, 1)$
6,449,436
107,838
122,055
$\mu_1 < 10^{-3}$ $\mu_2 \in [1,10)$
137,456
440
28
$\mu_1 \in [10^{-3}, 10^{-2})$ $\mu_2 \in [1,10)$
1,238,240
5,434
6,034
$\mu_1 \in [10^{-2}, 10^{-1})$ $\mu_2 \in [1,10)$
1,760,232
57,397
73,413
$\mu_1 \in [10^{-1}, 1)$ $\mu_2 \in [1,10)$
1,020,128
187,037
198,158
$\mu_1 \in [1,10)$ $\mu_2 \in [1,10)$
40,186
25,783
26,122
$\mu_1 < 10^{-3}$ $\mu_2 > 10$
7,260
120
0
$\mu_1 \in [10^{-3}, 10^{-2})$ $\mu_2 > 10$
65,400
1,585
1,385
$\mu_1 \in [10^{-2}, 10^{-1})$ $\mu_2 > 10$
92,970
11,757
13,341
$\mu_1 \in [10^{-1}, 1)$ $\mu_2 > 10$
53,880
25,252
26,292
$\mu_1 \in [1,10)$ $\mu_2 > 10$
4,260
3,710
3,733
$\mu_1 > 10$ $\mu_2 > 10$
105
105
105
$\mu_1 \in [0.1,1)$ $\mu_2 \in [1,10)$

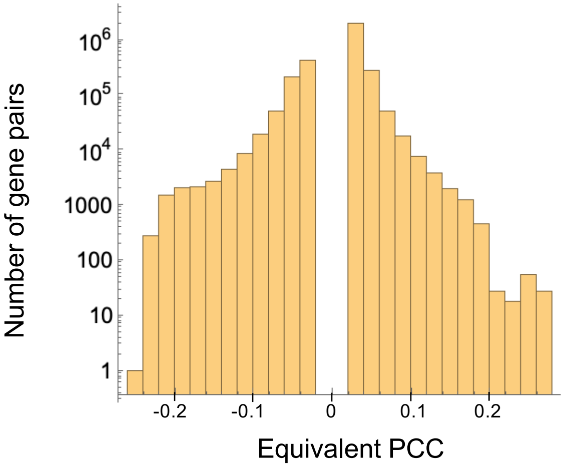

Number of gene pairs
10^6
10^5
10^4
1000
100
10
1
-0.2
-0.1
0
0.1
0.2
Equivalent PCC

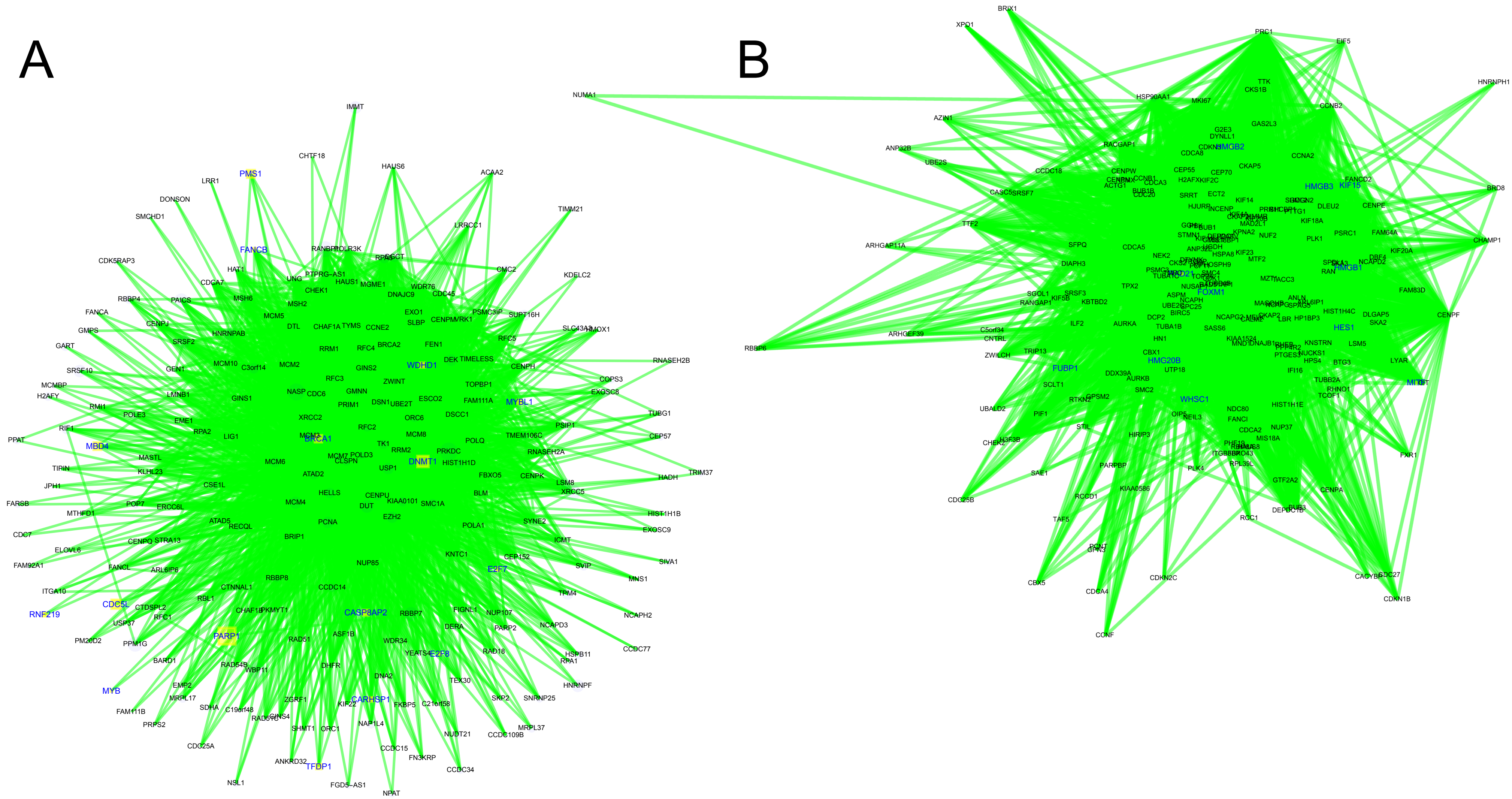

A
B
IMMT
CHTF18
PMS1
LRR1
HAUS6
ACAA2
DONSON
SMCHD1
TIMM21
LRRCC1
FANCB
RANBP1
POLR3K
RPA3
CCT
CDK5RAP3
HAT1
CMC2
KDELC2
CDCA7
UNG
PTPRG-AS1
HAUS1
MGME1
WDR76
CHEK1
DNAJC9
CDC45
RBBP4
PAICS
MSH6
MSH2
PSMC3IP
SUPT16H
FANCA
MCM5
EXO1
CENPM
VRK1
CENPJ
DTL
CHAF1A
TYMS
CCNE2
SLBP
GMPS
HNRNPAB
SLC43A3
HMOX1
SRSF2
RFC5
GART
RRM1
RFC4
BRCA2
FEN1
MCM10
C3orf14
MCM2
GINS2
WDHD1
DEK
TIMELESS
RNASEH2B
SRSF10
GEN1
CENPH
RFC3
ZWINT
TOPBP1
COPS3
MCMBP
LMNB1
GMNN
NASP
CDC6
H2AFY
GINS1
PRIM1
DSN1
UBE2T
ESCO2
FAM111A
MYBL1
EXOSC8
RMI1
POLE3
EME1
XRCC2
ORC6
DSCC1
TUBG1
RIF1
RPA2
RFC2
PSIP1
LIG1
MCM3
BRCA1
MCM8
TMEM106C
CEP57
PPAT
MBD4
TK1
POLQ
MASTL
MCM7
POLD3
RRM2
PRKDC
RNASEH2A
CLSPN
TIPIN
MCM6
DNMT1
HIST1H1D
TRIM37
KLHL23
USP1
ATAD2
FBXO5
CENPK
HADH
JPH1
CSE1L
LSM8
HELLS
BLM
XRCC5
FARSB
POP7
CENPU
MCM4
DUT
KIAA0101
SMC1A
ERCC6L
MTHFD1
EZH2
HIST1H1B
ATAD5
PCNA
POLA1
SYNE2
RECQL
CDC7
EXOSC9
BRIP1
CENPQ
STRA13
ICMT
ELOVL6
KNTC1
CEP152
FANCL
NUP85
FAM92A1
SVIP
E2F7
SIVA1
ARL6IP6
RBBP8
CTNNAL1
MNS1
ITGA10
CCDC14
TPM4
RBL1
CDC5L
CTDSPL2
FIGNL1
NCAPH2
RFC1
CHAF1B
PKMYT1
CASP8AP2
RBBP7
NUP107
RNF219
USP37
NCAPD3
PARP2
DERA
PARP1
PM20D2
PPM1G
RAD51
ASF1B
WDR34
RAD18
CCDC77
YEATS4
E2F8
HSPB11
BARD1
RPA1
RAD54B
WBP11
DHFR
DNA2
TEX30
HNRNPF
MYB
EMP2
MRPL17
SKP2
SNRNP25
SDHA
C19orf48
ZGRF1
CARHSP1
FKBP5
C21orf58
KIF22
FAM111B
RAD51C
GINS4
NAP1L4
MRPL37
PRPS2
SHMT1
ORC1
NUDT21
CCDC109B
CDC25A
CCDC15
FN3KRP
ANKRD32
TFDP1
CCDC34
NSL1
FGD5-AS1
NPAT
NUMA1
BRIX1
XPO1
PRC1
EIF5
HNRNPH1
TTK
CKS1B
HSP90AA1
MKI67
CCNB2
AZIN1
GAS2L3
G2E3
DYNLL1
CDKN3
HMGB2
ANP32B
RACGAP1
CDCA8
CCNA2
UBE2S
CCDC18
CKAP5
CEP55
CEP70
CENPW
CENPN
H2AFX
KIF2C
FANCD2
HMGB3
KIF15
CCNB1
CDCA3
ACTG1
BUB1B
CASC5
SRSF7
CDC20
SRRT
ECT2
BRD8
KIF14
HJURP
INCENP
DLEU2
CENPE
MAD2L1
KIF18A
TTF2
KPNA2
NUF2
PLK1
PSRC1
FAM64A
STMN1
BUB1
ARHGAP11A
SFPQ
CDCA5
ANP32E
HSPA8
KIF23
CHAMP1
KIF20A
NEK2
MTF2
DBF4
NCAPD2
DIAPH3
PSMC3
SKA3
HMGB1
RAN
HOSPH9
TUBA1C
TOP2A
SMC4
MZT1
TACC3
TPX2
NUSAP1
FOXM1
FAM83D
ASPM
SGOL1
KIF5B
SRSF3
NCAPH
ANLN
ARL6IP1
RANGAP1
KBTBD2
UBE2C
SPC25
HIST1H4C
CENPF
BIRC5
NCAPG2
DCP2
CKAP2
LBR
HP1BP3
DLGAP5
C5orf34
ILF2
AURKA
TUBA1B
SASS6
SKA2
ARHGEF39
HES1
CNTRL
KIAA1524
HN1
KNSTRN
LSM5
RBBP6
TRIP13
PTGES3
NUCKS1
ZWILCH
CBX1
HPS4
BTG3
LYAR
HMG20B
UTP18
FUBP1
IFI16
DDX39A
AURKB
TUBB2A
SCLT1
RHNO1
MITF
GPSM2
SMC2
TCOF1
RTKN2
WHSC1
UBALD2
HIST1H1E
PIF1
NEIL3
NDC80
FANCI
CDCA2
STIL
NUP37
H3F3B
HIRIP3
MIS18A
CHEK2
PHF19
ITGB3BP
FBXO43
FXR1
RPL39L
SAE1
PARPBP
PLK4
GTF2A2
KIAA0586
CENPA
CDC25B
RCCD1
BUB3
DEPDC1B
TAF5
RCC1
PCNT
GPN3
CACYBP
CDC27
CBX5
CDKN2C
CDCA4
CDKN1B
CCNF

C

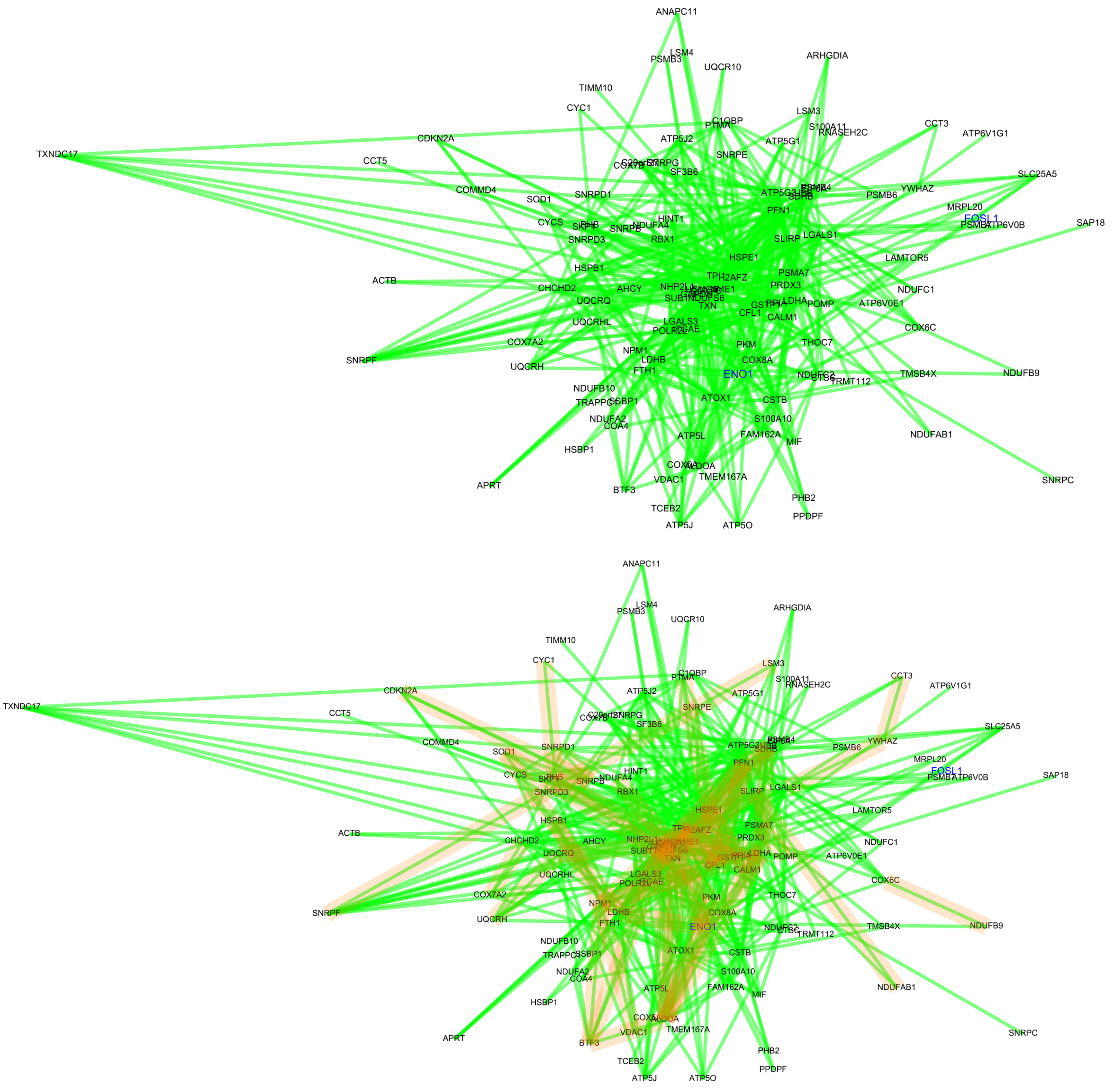
ANAPC11
LSM4
PSMB3
UQCR10
ARHGDIA
TIMM10
CYC1
LSM3
C1QBP
PTMA
S100A11
RNASEH2C
CCT3
ATP6V1G1
CDKN2A
ATP5J2
ATP5G1
TXNDC17
CCT5
SNRPE
SF3B6
SLC25A5
COMMD4
SOD1
SNRPD1
PSMB6
YWHAZ
MRPL20
FOSL1
SAP18
PFN1
CYCS
HINT1
NDUFA4
SNRPB
RBX1
SNRPD3
SLIRP
LGALS1
HSPE1
LAMTOR5
HSPB1
PSMA7
ACTB
CHCHD2
AHCY
PRDX3
NDUFC1
UQCRQ
TXN
CFL1
LDHA
POMP
ATP6V0E1
CALM1
UQCRHL
LGALS3
COX6C
COX7A2
THOC7
PKM
SNRPF
NPM1
COX8A
UQCRH
LDHB
FTH1
ENO1
TMSB4X
NDUFB9
TRMT112
NDUFB10
ATOX1
CSTB
S100A10
NDUFA2
COA4
FAM162A
NDUFAB1
ATP5L
MIF
HSBP1
APRT
SNRPC
TMEM167A
VDAC1
BTF3
PHB2
TCEB2
PPDPF
ATP5J
ATP5O

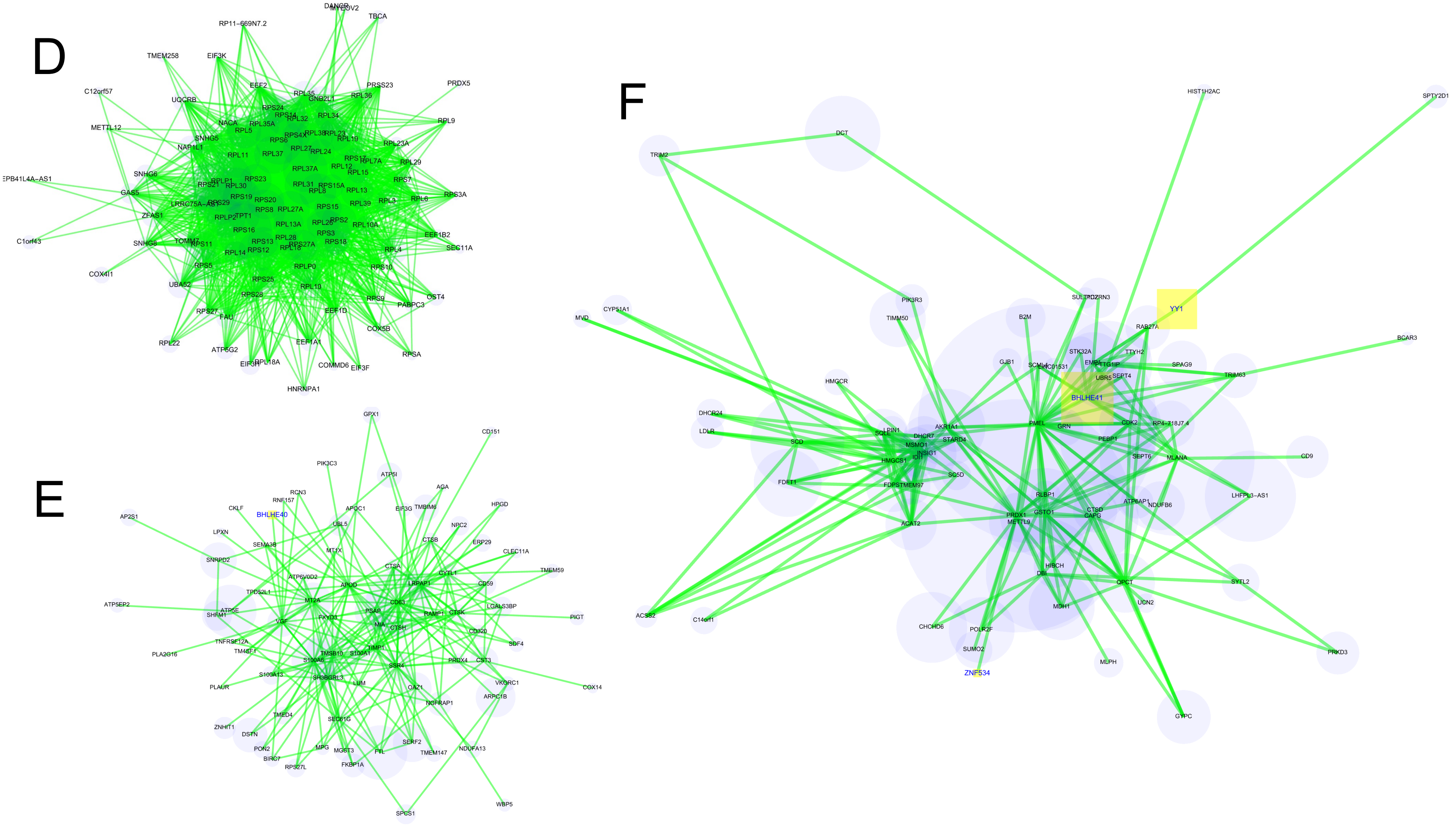

D
E
F
RP11-669N7.2
TBCA
TMEM258
EIF3K
PRDX5
C12orf57
EEF2
PRSS23
UQCRB
RPL35
GNB2L1
RPL36
METTL12
RPS24
RPL34
RPL9
NACA
RPL35A
RPL32
RPL5
RPS4X
RPL38
RPL23
NAP1L1
SNHG5
RPS6
RPL19
RPL23A
RPL27
RPL24
RPL11
RPL37
RPS17
RPL7A
RPL29
SNHG6
RPL37A
RPL12
RPL15
RPS7
RPS23
RPL31
RPS15A
RPL8
RPL13
RPS3A
GAS5
RPS21
RPL30
RPL1
RPS19
RPS20
RPL39
RPL3
RPL6
LRRC75A-AS1
RPS29
RPS8
RPL27A
RPS15
ZFAS1
RPLP2
TPT1
RPS2
RPL10A
RPS16
RPL13A
RPL26
EEF1B2
C1orf43
SNHG8
TOMM7
RPS11
RPS13
RPS3
RPS18
SEC11A
RPL28
RPS27A
RPL18
RPL14
RPS12
RPL4
RPS5
COX4I1
RPLP0
RPS10
RPS25
UBA52
RPL10
OST4
RPS28
RPS9
PABPC3
RPS27
EEF1D
FAU
COX5B
RPL22
ATP5G2
EEF1A1
RPSA
EIF3H
RPL18A
COMMD6
EIF3F
HNRNPA1
GPX1
CD151
PIK3C3
ATP5I
AGA
RCN3
RNF157
TMBIM6
HPGD
CKLF
BHLHE40
APOC1
EIF3G
AP2S1
UBL5
NPC2
LPXN
CTSB
ERP29
SEMA3B
MT1X
CLEC11A
SNRPD2
CTSA
CYTL1
TMEM59
ATP6V0D2
TPD52L1
APOD
LRPAP1
CD59
ATP5EP2
MT2A
CD63
LGALS3BP
ATP5E
SHFM1
PSAP
PIGT
FXYD3
RAMP1
CTSK
VGF
MIA
CTSH
CD320
TNFRSF12A
TIMP1
SDF4
TM4SF1
PLA2G16
TMSB10
S100A1
S100A6
PRDX4
CST3
S100A13
SSR4
SH3BGRL3
VKORC1
PLAUR
LUM
OAZ1
COX14
ARPC1B
NGFRAP1
TMED4
ZNHIT1
SEC61G
DSTN
SERF2
PON2
MPG
MGST3
FTL
TMEM147
NDUFA13
BIRC7
RPS27L
FKBP1A
SPCS1
WBP5
HIST1H2AC
SPTY2D1
DCT
TRIM2
PIK3R3
SULT1C2
PDZRN3
YY1
CYP51A1
TIMM50
B2M
MVD
RAB27A
BCAR3
STK32A
TTYH2
GJB1
SCML4
LINC01531
EMP1
PTTG1IP
SPAG9
HMGCR
UBR5
SEPT4
TRIM63
BHLHE41
DHCR24
AKR1A1
PMEL
GRN
CDK2
RP4-718J7.4
LPIN1
SQLE
DHCR7
LDLR
STARD4
PEBP1
SCD
MSMO1
INSIG1
CD9
HMGCS1
IDI1
SEPT6
MLANA
SC5D
FDFT1
FDPS
TMEM97
LHFPL3-AS1
RLBP1
ATP6AP1
NDUFB6
CTSD
PRDX1
GSTO1
CAPG
METTL9
ACAT2
HIBCH
DBI
QPCT
SYTL2
ACSS2
MDH1
UCN2
C14orf1
CHCHD6
POLR2F
PRKD3
SUMO2
MLPH
ZNF534
GYPC

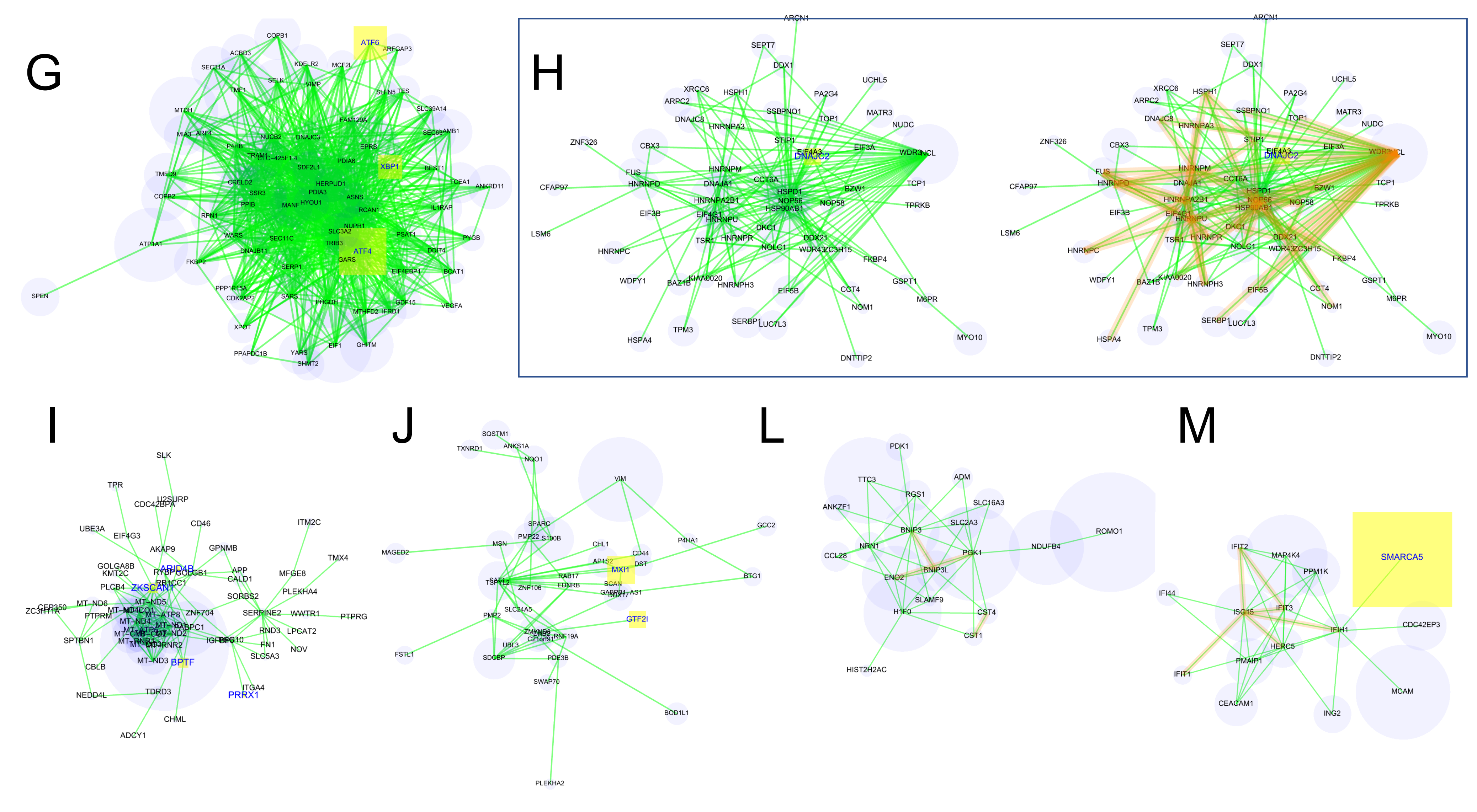
G
COPB1
ATF6
ARFGAP3
ACBD3
KDELR2
MCF2L
SEC31A
SELK
VIMP
TMF1
SLFN5
TES
MTDH
SLC39A14
FAM129A
MIA3
ARF4
NUCB2
DNAJC3
LAMB1
P4HB
EPRS
TRAM1
XBP1
BEST1
SDF2L1
PDIA6
TMED9
CRELD2
HERPUD1
TCEA1
ANKRD11
COPB2
SSR3
PDIA3
ASNS
PPIB
MANF
HYOU1
IL1RAP
RPN1
RCAN1
NUPR1
WARS
SLC3A2
PSAT1
PYGB
ATP1A1
SEC11C
TRIB3
DNAJB11
ATF4
DDIT4
FKBP2
GARS
SERP1
EIF4EBP1
BCAT1
SPEN
PPP1R15A
CDK2AP2
SARS
PHGDH
GDF15
VEGFA
MTHFD2
IFRD1
XPOT
YARS
EIF1
GHITM
PPAPDC1B
SHMT2
H
ARCN1
SEPT7
DDX1
UCHL5
XRCC6
HSPH1
PA2G4
ARPC2
SSBPNO1
MATR3
DNAJC8
TOP1
NUDC
HNRNPA3
ZNF326
CBX3
STIP1
EIF4A3
DNAJC2
EIF3A
WDR3
NCL
FUS
HNRNPM
CFAP97
HNRNPD
DNAJA1
CCT6A
TCP1
BZW1
HSPD1
NOP56
NOP58
HNRNPA2B1
HSP90AB1
TPRKB
EIF3B
EIF4G1
HNRNPU
LSM6
DKC1
TSR1
HNRNPR
DDX21
HNRNPC
NOLC1
WDR43
ZC3H15
FKBP4
WDFY1
KIAA0020
BAZ1B
HNRNPH3
GSPT1
EIF5B
CCT4
M6PR
NOM1
SERBP1
LUC7L3
TPM3
HSPA4
MYO10
DNTTIP2
I
SLK
TPR
U2SURP
CDC42BPA
CD46
ITM2C
UBE3A
EIF4G3
AKAP9
GPNMB
TMX4
GOLGA8B
ARID4B
KMT2C
APP
MFGE8
CALD1
PLCB4
ZKSCAN1
RB1CC1
PLEKHA4
SORBS2
MT-ND6
MT-ND5
ZNF704
SERPINE2
WWTR1
PTPRG
PTPRM
MT-ND4
MT-ATP8
RND3
LPCAT2
SPTBN1
FN1
NOV
SLC5A3
CBLB
MT-ND3
BPTF
ITGA4
NEDD4L
TDRD3
PRRX1
CHML
ADCY1
J
SQSTM1
TXNRD1
ANKS1A
NQO1
VIM
GCC2
SPARC
PMP22
S100B
P4HA1
MAGED2
MSN
CHL1
CD44
DST
MXI1
BTG1
RAB17
ZNF106
EDNRB
BCAN
SLC24A5
GTF2I
PMP2
RNF19A
FSTL1
UBL3
SDCBP
PDE3B
SWAP70
BOD1L1
PLEKHA2
L
PDK1
TTC3
ADM
RGS1
SLC16A3
ANKZF1
BNIP3
SLC2A3
NRN1
PGK1
NDUFB4
ROMO1
CCL28
BNIP3L
ENO2
SLAMF9
H1F0
CST4
CST1
HIST2H2AC
M
IFIT2
MAP4K4
SMARCA5
PPM1K
IFI44
ISG15
IFIT3
CDC42EP3
IFIH1
HERC5
PMAIP1
IFIT1
CEACAM1
ING2
MCAM
